# The Effect of Acupuncture on the Quality of Life in Patients With Migraine: A Systematic Review and Meta-Analysis

**DOI:** 10.3389/fphar.2018.01190

**Published:** 2018-10-26

**Authors:** Yin Jiang, Peng Bai, Hao Chen, Xiao-Yu Zhang, Xiao-Yi Tang, He-Qing Chen, Ye-Yin Hu, Xiao-Li Wang, Xin-Yi Li, You-Ping Li, Gui-Hua Tian

**Affiliations:** ^1^Dongzhimen Hospital, Beijing University of Chinese Medicine, Beijing, China; ^2^The Second Clinical College, Nanjing University of Chinese Medicine, Nanjing, China; ^3^Chinese Cochrane Center, West China Hospital, Sichuan University, Chengdu, China

**Keywords:** migraine, acupuncture, systematic review, meta-analysis, efficacy and safety, adverse events, quality of life

## Abstract

**Background:** Acupuncture is frequently used as an efficient method to prevent and treat migraines. However, its effect on the quality of life remains controversial.

**Methods:** Seven databases, such as PubMed and Cochrane Library were searched to retrieve reference lists of eligible trials and related reviews. Randomized controlled trials that were published in Chinese and English were included.

**Results:** Acupuncture resulted in lower Visual Analog Scale scores than the medication group at 1 month after treatment (MD −1.22, 95%CI −1.57 to −0.87; low quality) and 1–3 months after treatment (MD −1.81, 95%CI −3.42 to −0.20; low quality). Compared with sham acupuncture, acupuncture resulted in lower Visual Analog Scale scores at 1 month after treatment (MD −1.56, 95%CI −2.21 to −0.92; low quality).

**Conclusion:** Acupuncture exhibits certain efficacy both in the treatment and prevention of migraines, which is superior to no treatment, sham acupuncture and medication. Further, acupuncture enhanced the quality of life more than did medication.

## Introduction

Migraine, a common neurological disorder, is the third most prevalent disease worldwide out of all medical conditions and the seventh highest cause of disability according to the Global Burden of Disease Study (Vos et al., 2012; Steiner et al., 2013; Diener et al., 2015; Rodriguez, 2015). In China, the prevalence of migraine is 9.3%, with annual expenses of 331.7 billion yuan (Steiner et al., 2013). Thus, migraine has broad effects and a significant social and economic burden (Yu et al., 2012). The recurrent and refractory pain, accompanied by long-term economic stress, may aggravate psychological pressure of patients and cause further tension and anxiety. Migraine is also affected by psychological factors, such as stress and emotion; therefore, a vicious circle is created, which is detrimental for treatment. In addition, migraine attacks affect daily work and life, even leaving some patients unable to work and live independently. Therefore, the quality of life in migraine patients' needs further attention.

Migraines are typically treated by various medications or nonpharmacotherapy to relieve pain or prevent attacks (Holland et al., 2012; Silberstein et al., 2012a; group CMAopchp, 2016). However, those drugs have limited efficacy in relieving headache or reducing the frequency of attacks and are often accompanied by adverse effects (Cranz, 1990; Lipton et al., 1994; Silberstein and Young, 1995; Silberstein et al., 2012b). Acupuncture has become a widely used complementary therapy in many countries (Hartel and Volger, 2004; Bodeker et al., 2005; Burke et al., 2006). Due to its stable effects, acupuncture has been increasingly used in migraine prevention. Some studies have reported that compared with medication, acupuncture had similar or even better effects in raising efficiency rates and reducing migraine attacks (Linde et al., 2009, 2016; Gao et al., 2011; Zheng and Cui, 2012; Song et al., 2016). It has also been reported that acupuncture had fewer side effects and better tolerance (Higgins and Green, 2011; Sterne et al., 2011; Gao, 2012). Therefore, due to its safety and efficacy, acupuncture is expected to be an important method for the prevention and treatment of migraine.

However, some clinical trials have found that there was little or no difference in the preventive effects between acupuncture and sham acupuncture (Linde et al., 2005; Li et al., 2012; Rodriguez, 2015). A prior study even indicated that the main effects of acupuncture may come from placebo effects (Meissner et al., 2013). Thus, the efficacy of acupuncture on migraine needs further verification. In addition, some studies have shown that acupuncture can improve depressive symptoms caused by migraine and observed post-stroke, but the efficacy in relieving psychological stress is still unclear.

Hence, the purpose of this analysis was to evaluate the efficacy and safety of acupuncture for the treatment of migraine. Further, through this research, we also aimed to confirm the effect of acupuncture for improving anxiety.

## Materials and methods

### Systematic review details

The systematic review was performed in accordance with the Cochrane Handbook for Systematic Reviews of Interventions and was reported in compliance with the PRISMA statement (see Supplementary PRISMA 2009 Checklist).

### Search strategy

We searched the following 7 databases from inception to Oct 19th, 2017: PubMed, the Cochrane Library, Web of Science, EMBASE, China Biology Medicine disc (CBM), China National Knowledge Infrastructure (CNKI) and the Wanfang Database. Details of the search strategies are available in the Supplementary Search Terms and Strategies. We also searched the references from the included literature and related systematic reviews to identify further studies that met the inclusion criteria for this review.

### Inclusion criteria

#### Types of studies

Randomized control trials (RCTs) on acupuncture therapy for migraine were included. Quasi-randomized controlled trials (Quasi-RCTs) were excluded. The follow-up time was not limited. And studies meeting the criteria should be included without language and publish limits.

#### Types of participants

Participants were diagnosed as migraine with aura, without aura or other special types, according to the International Classification of Headache Disorders (ICHD-II) (Jes, 2004). Patients were excluded if the migraine had been caused by organic disorders (such as subarachnoid hemorrhage, cerebral hemorrhage, cerebral embolism, cerebral thrombosis, vascular malformation, arteritis, hypertension, arteriosclerosis, etc.).

### Types of interventions

The intervention that we were concerned with in this review was acupuncture therapy. Any treatment that utilized filiform needles to prick acupoints, Ashi points or meridian locations was regarded as acupuncture therapy. Studies based on “microsystems” theory, such as eye-acupuncture and ear-acupuncture, were excluded. Acupoint injection was also excluded.

For included studies, the control group received no treatment, sham acupuncture or medication. No treatment meant that the patients did not receive acupuncture or any other treatment throughout the duration of the study, except when necessary to treat acute attacks. Sham acupuncture was performed without skin penetration, utilizing incorrect acupoint locations, or both. Medication refers to drugs recommended by clinical guidelines, such as calcium antagonists, antiepileptic drugs, and triptans. Traditional Chinese medicine, Chinese patent medicine and non-recommended drugs, such as Nimodipine were excluded. In addition, trials with combined therapies were not included.

### Types of outcome measures

The primary outcomes that were selected consisted of the Visual Analog Scale (VAS, from 0 to 10 scores), frequency of migraine attacks (numbers/month), Migraine-Specific Quality of Life Questionnaire (MSQ), and Self-rating Depression Scale (SDS). Secondary outcomes consisted of days of migraine (days/month), adverse events (numbers of adverse event attacks), and total effective rate, which was reported as the percentage of the total participants that showed symptom improvement ≥20%.

### Study selection

The reference management software Endnote X7 was used to remove the duplicate records. Two reviewers (H-QC and XL) screened the titles and abstracts of all identified studies for relevance and labeled records as included, excluded, or uncertain. Full-text articles were obtained for assessing eligibility in the case of uncertainty. If studies lacked key information, the reviewers sent emails to the author for further information. If the author did not respond, literature that lacked information was excluded. Disagreements were resolved by the third reviewer (X-YT).

### Data extraction

Two reviewers (XW Wang and YH) independently extracted the data. We collected the following information using a standard form: general information (sample size, age, gender and accurate diagnosis), details of the intervention and comparison (study types, duration, treatment frequency, medication dosage, etc.), and the outcomes. Disagreements were resolved by the third reviewer.

### Risk of bias assessment

Two reviewers (H-QC and YH) independently assessed the risk of bias using the tool recommended by the Cochrane Collaboration (Higgins and Green, 2011). Each trial was scored as having high, low or unclear risk for the following 7 domains: (1) random sequence generation (selection bias); (2) allocation concealment (selection bias); (3) blinding of participants and personnel (performance bias); (4) blinding of outcome assessment (detection bias); (5) incomplete outcome data (attrition bias); (6) selective reporting (reporting bias); and (7) any other bias. Disagreements were resolved in consultation with the third reviewer (GT).

### Statistical analysis

Meta-analysis was performed using RevMan 5.3 software. Continuous data were presented as the mean differences (MDs) with a 95% confidence interval (CI), whereas dichotomous data were presented as relative risk (RR) with a 95% CI. Statistical heterogeneity across trials was assessed by the Cochrane Q test (*P* < 0.1 for statistical significance) and quantified by the *I*^2^ statistic. An *I*^2^ >50% indicated significant heterogeneity. Heterogeneous data were pooled using the random-effects model. We performed the analysis based on the time window, which we defined as (1) immediate effects (≤30 min after one treatment); (2) up to 1 month after treatment; (3) 1–3 months after treatment; (4) 3–6 months after treatment; and (5) >6 months after treatment. Subgroup analysis was performed based on adequacy of concealment (unambiguously adequate concealment vs. none or unclear adequacy); sample size (≥median sample vs. < median sample); treatment time (≥1 month vs. < 1 month); sham acupuncture methods (deep insertion at nonacupoints vs. superficial insertion at acupoints vs. superficial insertion at nonacupoints), and types of medication. In addition, to investigate potential sources of heterogeneity in our findings, we performed subgroup analyses. Further sensitivity analysis was conducted to test the impact of the quality of included trials, if needed.

### Assessment of reporting biases

Publication bias was evaluated by visual inspection of a funnel plot if we included at least 10 trials in the comparison (Sterne et al., 2011; Chang et al., 2012).

## Results

### Study selection

**Figure 1:**
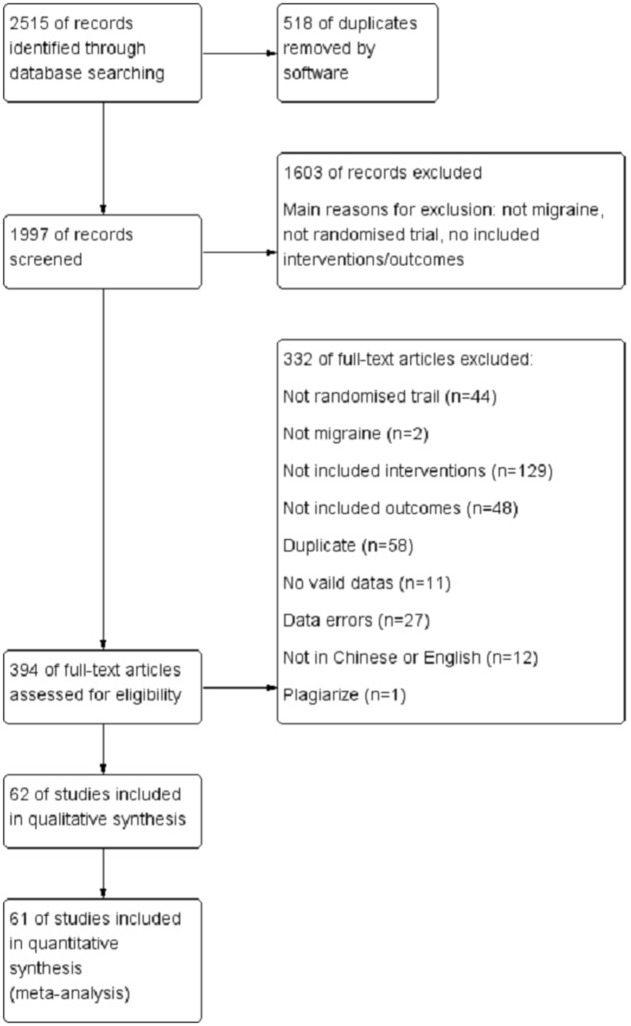
Flow chart.

Figure 1 shows a flow chart of the study selection process according to PRISMA guideline. The initial search yielded 2,515 records, of which 518 records were removed for duplication. After screening the titles and abstracts, 394 were deemed to potentially be eligible. After reviewing the full text, 332 records were excluded (2 had patients who were not diagnosed as migraine; 129 used interventions or comparisons that did not meet the standards; 48 measured outcomes that were not included; 44 were not RCTs; 58 were repeatedly published; 11 were conference papers, protocols or other papers without valid data; 27 had data errors; 12 were not published in Chinese or English; and 1 was suspected as being plagiarized). In total, 62 trials were included for the final analysis.

### Study characteristics

The characteristics of the included studies are summarized in Supplementary Table 1. The 62 studies included 4,947 participants (median number of participants 64.5, range 22–302); 50 trials recruited participants from China, 3 from Brazil, 3 from Germany, 2 from Italy and others from Iran, Israel, Australia and Sweden. For age distribution, 57 studies were performed on adults, 2 on adults and the elderly, 1 on adolescents, and 2 on patients without a reported age range. A total of 41 studies included medication as the control group, 22 included sham acupuncture, and 1 included no treatment. Two studies had three groups for comparison. The main acupoints selected were Shuaigu (GB8), Fengchi (GB20), Yanglingquan (GB34), Zulinqi (GB41), Baihui (GV20), Yintang (GV29), Waiguan (SJ5), Sizhukong (SJ23), and Hegu (LI4). In most studies the treatment time was 1 month (21 trials), 21 trials lasted < 1 month, 3 had a duration of 2 months, 8 had a duration of 3 months, 2 had a duration of 6 months, 6 were evaluated for immediate effects, and only 1 had an unclear treatment time. The treatment frequency was mainly once a day. The follow-up times were reported to be < 1 year, with most focused on 1 month after treatment.

### Risk of bias within studies

**Figure 2:**
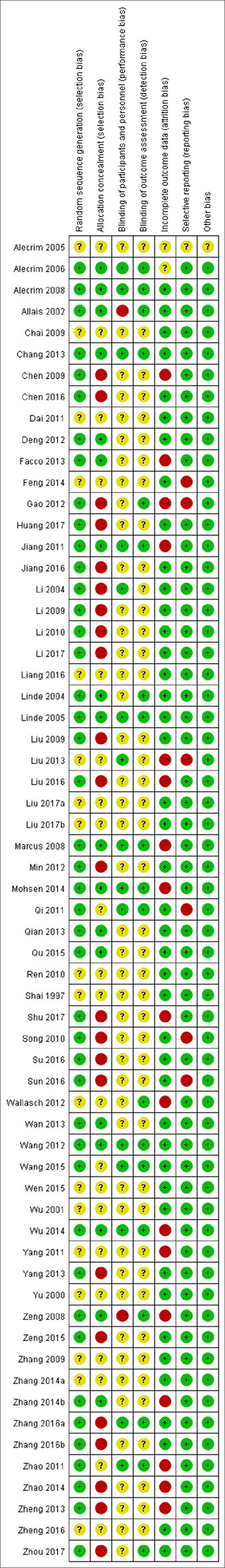
Risk of bias graph.

As presented in Figure 2, most of the included studies were evaluated as having a high risk of bias based on the Cochrane risk of bias tool. There were 23 studies that were at a high risk of bias in allocation concealment, 3 at a high risk of bias in blinding toward participants, 1 at a high risk of bias in blinding of the outcome assessors, 16 at a high risk of bias in attrition bias, and 6 at a high risk of bias in selective reporting bias.

### Effects of interventions

#### Primary outcomes

##### VAS scores

**Figure 3:**
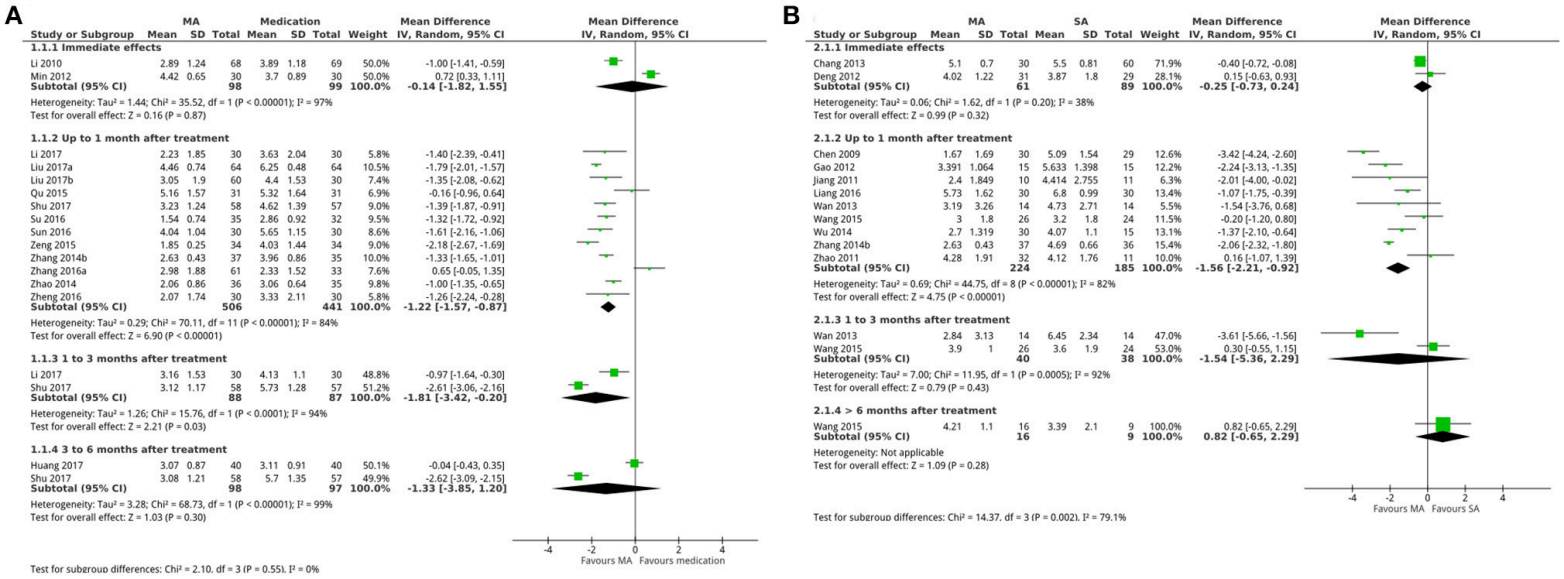
Forest plot: **(A)** acupuncture vs. medication- VAS Scores; **(B)** sham acupuncture- VAS scores. MA, manual acupuncture; SA, sham acupuncture.

A total of 12 studies that included 947 patients reported VAS scores for acupuncture and medication (Zhang, 2014; Zhao, 2014b; Qu and Shen, 2015; Zeng and Li, 2015; Su et al., 2016; Sun et al., 2016; Zhang X., 2016; Zheng, 2016; Li, 2017; Liu, 2017; Liu et al., 2017; Shu et al., 2017). The pooled results indicated that VAS scores were lower with acupuncture than with medication at a follow-up time of up to 1 month after treatment (random-effects estimates; MD −1.22, 95%CI −1.57 to −0.87; *P*-value of the Chi^2^ test <0.00001, *I*^2^ = 84%; *P*-value of Z test <0.00001; low quality; Figure 3A shows the results). While 2 studies that included 175 patients reported VAS scores that were lower in acupuncture compared with medication at a follow-up time of 1–3 months after treatment (random-effects estimates; MD −1.81, 95%CI −3.42 to −0.20; *P*-value of the Chi^2^ test <0.0001, *I*^2^ = 94%; *P*-value of Z test = 0.03; very low quality; Figure 3A shows the results; Li, 2017; Shu et al., 2017). For acupuncture and sham acupuncture,a total of 9 trials that included 409 patients presented VAS scores were lower with acupuncture than with sham acupuncture at a follow-up time of up to 1 month after treatment (random-effects estimates; MD −1.56, 95%CI −2.21 to −0.92; *P*-value of the Chi^2^ test <0.00001, *I*^2^ = 82%; *P*-value of Z test <0.00001; low quality; Figure 3B shows the results; Chen, 2009; Jiang, 2011; Jiang et al., 2011; Zhao, 2011; Gao, 2012; Wan et al., 2013; Wu, 2014; Zhang, 2014; Wang et al., 2015; Liang et al., 2016).

##### MSQ scores

**Figure 4:**
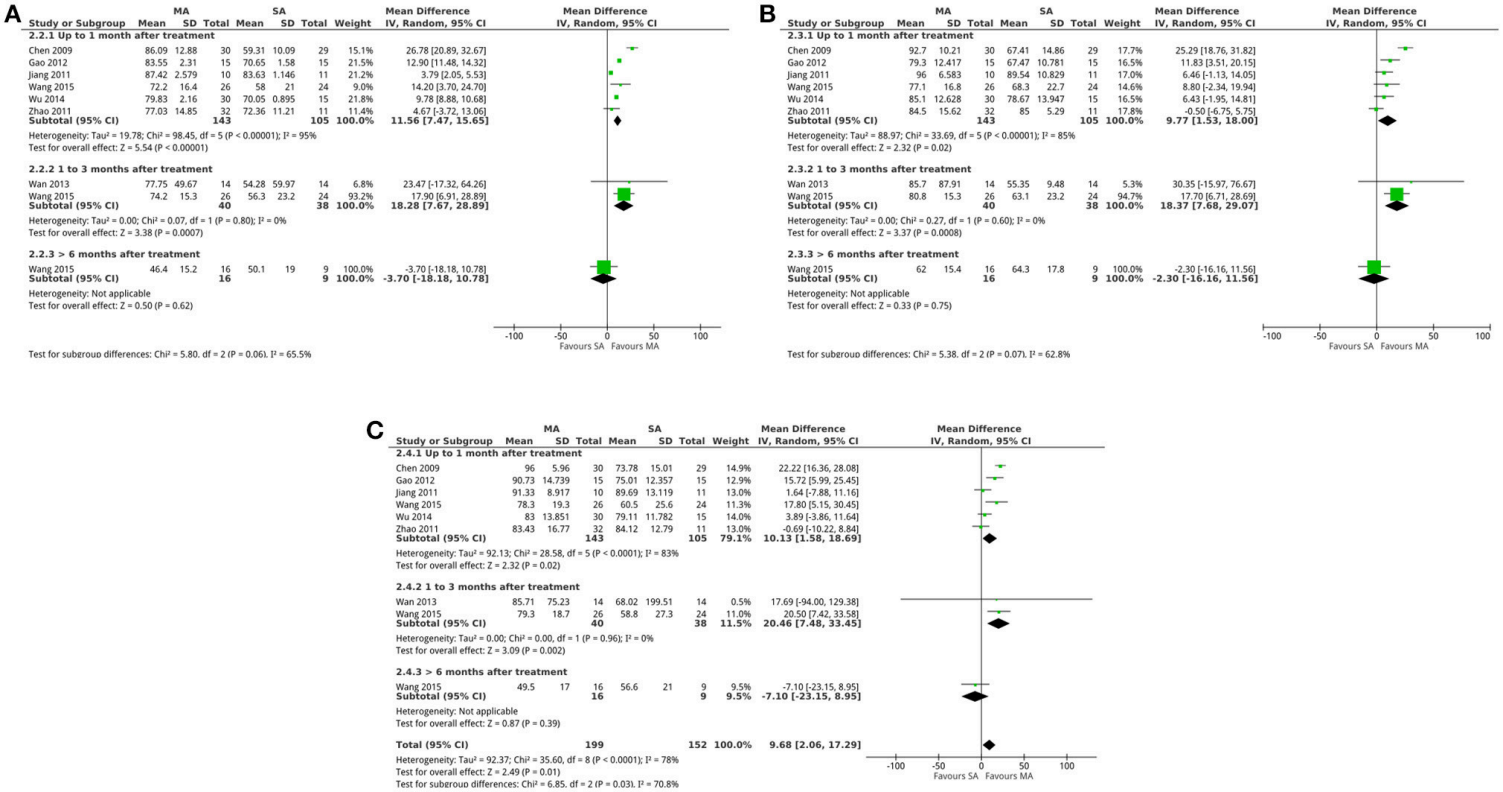
Forest plot: **(A)** acupuncture vs. sham acupuncture- MSQ scores: **(A)** role function-restrictive; **(B)** role function-preventive; **(C)** emotional function. MA, manual acupuncture; SA, sham acupuncture.

The pooled results of 6 trials involving 248 patients showed MSQ scores that were greater with acupuncture than sham acupuncture at a follow-up time of up to 1 month after treatment (Chen, 2009; Jiang, 2011; Zhao, 2011; Gao, 2012; Wu, 2014; Wang et al., 2015), which included scores of role function-restrictive (random-effects estimates; MD 11.56, 95%CI 7.47–15.65; *P*-value of the Chi^2^ test <0.00001, *I*^2^ = 95%; *P*-value of Z test <0.00001; low quality; Figure 4A shows the results), scores of role function-preventive (random-effects estimates; MD 9.77, 95%CI 1.53–18.00; *P*-value of the Chi^2^ test <0.00001, *I*^2^ = 85%; *P*-value of Z test = 0.02; very low quality; Figure 4B shows the results) and scores of emotional function (random-effects estimates; MD 10.13, 95%CI 1.58–18.69; *P*-value of the Chi^2^ test <0.00001, *I*^2^ = 83%; *P*-value of Z test = 0.02; very low quality; Figure 4C shows the results). Further, 2 trials that included 78 patients reported MSQ scores for acupuncture and sham acupuncture at a follow-up time of 1–3 months after treatment (Wan et al., 2013; Wang et al., 2015). The pooled results indicated that the MSQ scores were greater with acupuncture than with sham acupuncture, which include scores of role function-restrictive (random-effects estimates; MD 18.28, 95%CI 7.67–28.89; *P*-value of the Chi^2^ test = 0.80, *I*^2^ = 0%; *P*-value of Z test = 0.0007; high quality; Figure 4A shows the results), scores of role function-preventive (random-effects estimates; MD 18.37, 95%CI 7.68–29.07; *P*-value of the Chi^2^ test = 0.60, *I*^2^ = 85%; *P*-value of Z test = 0.0008; high quality; Figure 4B shows the results) and scores of emotional function (random-effects estimates; MD 20.46, 95%CI 7.48–33.45; *P*-value of the Chi^2^ test = 0.96, *I*^2^ = 0%; *P*-value of Z test = 0.002; high quality; Figure 4C shows the results).

##### Frequency of migraine attacks

Only 1 trial involving 221 patients, compared with no treatment group, reported attack frequency for up to 1 month after treatment (Linde et al., 2006). The results indicated that the attack frequency was less in the acupuncture group than in the no treatment group (MD −0.80, 95%CI −1.12 to −0.48; *P*-value of Z test <0.00001; Supplementary Table 2).

#### Secondary outcomes

##### Total effective rates

A total of 21 trials that included 1,598 patients showed total effective rates that were greater with acupuncture than medication at a follow-up time of up to 1 month after treatment (random-effects estimates; RR 1.19, 95%CI 1.13–1.27; *P*-value of the Chi^2^ test = 0.02, *I*^2^ = 43%; *P*-value of Z test <0.00001; low quality; Supplementary Figure A; Wu, 2001; Li and Jia, 2009; Liu, 2009; Zeng, 2009; Zhang, 2009, 2014; Song, 2010; Dai et al., 2011; Min, 2012; Qian and Wan, 2013; Zheng et al., 2013; Feng et al., 2014; Zhang and Huang, 2014; Zhao, 2014b; Wen, 2015; Chen, 2016; Jiang and Zheng, 2016; Liu and Yan, 2016; Sun et al., 2016; Zhang X., 2016; Li, 2017). The total effective rate was greater with acupuncture than medication in 6 trials that included 465 patients at a follow-up time of 3–6 months after treatment (random-effects estimates; RR 1.18, 95%CI 1.04–1.35; *P*-value of the Chi^2^ test = 0.06, *I*^2^ = 52%; *P*-value of Z test = 0.01; low quality; Supplementary Figure A; Song, 2010; Qi, 2011; Liu et al., 2013; Feng et al., 2014; Chen, 2016; Huang et al., 2017). One trial involving 70 patients presented total effective rates that were greater with acupuncture than medication at a follow-up time of more than 6 months (RR 1.43, 95%CI 1.11–1.85, *P*-value of Z test = 0.005; Supplementary Figure A; Chai, 2009).

There were three trials involving 180 patients who reported the total effective rate for acupuncture and sham acupuncture (Li, 2004; Chen, 2009; Zhang, 2014). The pooled results indicate that the total effective rate was greater with acupuncture than sham acupuncture at a follow-up time of up to 1 month after treatment (fixed-effect estimates; RR 2.43, 95%CI 1.82–3.23; *P*-value of the Chi^2^ test = 0.28, *I*^2^ = 22%; *P*-value of Z test <0.00001; low quality; Supplementary Figure F), while the results of 1 trial involving 48 patients presented total effective rates that were greater with acupuncture than sham acupuncture at 3–6 months after treatment (RR 5.21, 95%CI 1.75–15.49; *P*-value of Z test = 0.003; Supplementary Figure F; Li, 2004).

##### Days of migraine

One trial (Zhou, 2017) involving 64 patients reported that days of attack were 1.38 days/month less with acupuncture than with medication at a follow-up time of 1–3 months after treatment (MD −1.38, 95%CI −1.97 to −0.79; *P*-value of Z test <0.00001; Supplementary Figure B). 7 trials that included 488 patients reported days of attack for acupuncture and sham acupuncture at a follow-up time of up to 1 month after treatment (Alecrim-Andrade et al., 2005, 2006, 2008; Linde et al., 2006; Zhao, 2011; Wallasch et al., 2012; Wang et al., 2015). The pooled results indicated that days of attack were fewer with acupuncture compared with sham acupuncture (random-effects estimates; MD −1.30, 95%CI −2.45 to −0.16; *P*-value of the Chi^2^ test = 0.02, *I*^2^ = 61%; *P*-value of Z test = 0.03; low quality; Supplementary Figure G).

##### Adverse events

For adverse events, 14 studies (Yu et al., 2000; Allais et al., 2002; Zeng, 2009; Ren, 2010; Yang et al., 2012; Facco et al., 2013; Qian and Wan, 2013; Yang, 2013; Zheng et al., 2013; Zhao, 2014b; Qu and Shen, 2015; Zhang X., 2016; Zhang Y., 2016; Zhou, 2017) that included 1,245 participants showed that acupuncture had a lower risk than medication (random-effects estimates; RD −0.16, 95%CI −0.25 to −0.06, *P*-value of the Chi^2^ test <0.00001, *I*^2^ = 93%; *P*-value of Z test = 0.001; very low quality; Supplementary Figure C).

According to the meta-analysis results, the results of the VAS scores (immediate effects and 3–6 months after treatment; Figure 3A shows the results), attack frequency (up to 1 month after treatment; Supplementary Figure D), SDS scores (up to 1 month after treatment; Supplementary Figure E), total effective rates (1–3 months after treatment; Supplementary Figure A) and days of attack (up to 1 month after treatment; Supplementary Figure B) were similar for both acupuncture and medication groups. While VAS scores (immediate effects, 1–3 months after treatment and more than 6 months after treatment; Figure 3B shows the results), days of attack (more than 1 month after treatment; Supplementary Figure G), attack frequency (up to 6 months after treatment; Supplementary Figure H), MSQ (more than 6 months after treatment; Figure 4), SDS scores (up to 1 month after treatment; Supplementary Figure I), and adverse events (Supplementary Figure J) were similar for both acupuncture and sham acupuncture.

### Subgroup analysis

Following the meta-analysis, the comparison between acupuncture and medication indicated significant heterogeneity in terms of VAS scores, effective rate, days of attack and adverse events. Considering the clinical heterogeneity, we designed 4 subgroups for each outcome: sample size, treatment time, adequacy of concealment and type of medication. The results of this subgroup analysis are displayed in Supplementary Table 3. The subgroup analysis compared acupuncture and sham acupuncture for VAS scores (up to 1 month after treatment), attack frequency (up to 1 month, and 1–3 months after treatment), MSQ scores (up to 1 month after treatment) and days of attack (up to 1 month, and 1–3 months after treatment). The following covariates were selected for subgroup analysis: adequacy of concealment, sample size, treatment time and acupuncture methods. Detailed results are shown in Supplementary Table 4.

### Sensitivity analysis

**Figure 5:**
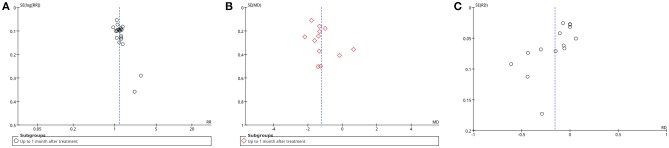
Funnel plot: **(A)** manual acupuncture vs. medication-effective rate-up to 1 month after treatment; **(B)** manual acupuncture vs. medication-VAS scores-up to 1 month after treatment; **(C)** manual acupuncture vs. medication-adverse events. Note: SE, standard error; RR, risk ratio.

This analysis also assessed reporting bias in acupuncture vs. medication via funnel plots of the following outcomes: VAS scores (up to 1 month after treatment), effective rate (up to 1 month after treatment), and adverse events. These 3 funnel plots were all asymmetric, so the assessors highly suspected the possibility of reporting bias. Additionally, it was not ruled out that the low methodological quality of the trials, specifically most having a small sample size, exaggerated the effects and caused the asymmetry (Figure 5).

After removing the trials causing asymmetry, a sensitivity analysis was performed for VAS scores (up to 1 month after treatment), and effective rate (up to 1 month after treatment). The detailed results of the sensitivity analysis for effective rate and VAS scores are shown in Table 1.

**Table 1:** Detailed information of trials assessed the sensitivity analysis.

**Excluded studies**	**Participants**	**MD (95% CI)**	***P*-value**	**Heterogeneity: *I*^2^**	**Effect model**
**VAS SCORES-UP TO 1 MONTH AFTER TREATMENT**					
Li, 2017	60	−1.21 [−1.57, −0.85]	*P* < 0.00001	86%	Random effects
Liu, 2017	128	−1.15 [−1.53,−0.78]	*P* < 0.00001	81%	Random effects
Liu et al., 2017	90	−1.21 [−1.58,−0.84]	*P* < 0.00001	86%	Random effects
Qu and Shen, 2015	62	−1.30 [−1.65,−0.96]	*P* < 0.00001	83%	Random effects
Shu et al., 2017	115	−1.20 [−1.58,−0.82]	*P* < 0.00001	86%	Random effects
Su et al., 2016	67	−1.21 [−1.59,−0.82]	*P* < 0.00001	86%	Random effects
Sun et al., 2016	60	−1.18 [−1.56,−0.81]	*P* < 0.00001	86%	Random effects
Zeng and Li, 2015	68	−1.13 [−1.48,−0.78]	*P* < 0.00001	83%	Random effects
Zhang, 2014	72	−1.20 [−1.60, −0.80]	*P* < 0.00001	86%	Random effects
Zhang X., 2016	94	−1.39 [−1.66,−1.12]	*P* < 0.00001	72%	Random effects
Zhao, 2014b	71	−1.24 [−1.62, −0.87]	*P* < 0.00001	84%	Random effects
Zheng, 2016	60	−1.22 [−1.58,−0.85]	*P* < 0.00001	86%	Random effects
**VAS SCORES-UP TO 1 MONTH AFTER TREATMENT**					
Chen, 2016	70	1.17 [1.12, 1.23]	*P* < 0.00001	18%	Random effects
Dai et al., 2011	40	1.17 [1.12, 1.23]	*P* < 0.00001	18%	Random effects
Feng et al., 2014	60	1.18 [1.12, 1.24]	*P* < 0.00001	17%	Random effects
Jiang and Zheng, 2016	92	1.17 [1.11, 1.22]	*P* < 0.00001	11%	Random effects
Li, 2017	60	1.18 [1.12, 1.24]	*P* < 0.00001	15%	Random effects
Liu, 2009	60	1.18 [1.12, 1.24]	*P* < 0.00001	17%	Random effects
Liu and Yan, 2016	180	1.19 [1.13, 1.25]	*P* < 0.00001	8%	Random effects
Qian and Wan, 2013	60	1.17 [1.12, 1.22]	*P* < 0.00001	13%	Random effects
Song, 2010	60	1.17 [1.12, 1.23]	*P* < 0.00001	17%	Random effects
Sun et al., 2016	60	1.17 [1.12, 1.23]	*P* < 0.00001	15%	Random effects
Wen, 2015	120	1.16 [1.11, 1.21]	*P* < 0.00001	0%	Random effects
Wu, 2001	63	1.17 [1.12, 1.23]	*P* < 0.00001	15%	Random effects
Zeng, 2009	64	1.18 [1.12, 1.24]	*P* < 0.00001	15%	Random effects
Zhang, 2009	60	1.17 [1.11, 1.23]	*P* < 0.00001	15%	Random effects
Zhang, 2014	80	1.17 [1.11, 1.23]	*P* < 0.00001	15%	Random effects
Zhang, 2014	72	1.18 [1.13, 1.24]	*P* < 0.00001	12%	Random effects
Zhang X., 2016	94	1.19 [1.13, 1.24]	*P* < 0.00001	0%	Random effects
Zhao, 2014b	71	1.17 [1.12, 1.23]	*P* < 0.00001	17%	Random effects
Zheng et al., 2013	117	1.17 [1.11, 1.22]	*P* < 0.00001	12%	Random effects

## Discussion

### Summary of main results

Acupuncture was more effective for short-term treatment compared with no treatment. Compared with medication, manual acupuncture resulted in better efficacy or safety, especially at follow-up times of up to 3 months after treatment, but it exhibited results with statistical heterogeneity. In the comparison between acupuncture and sham acupuncture, participants exhibited better pain relief and improved quality of life at follow-up times of up to 3 months after treatment, although with statistical heterogeneity. In the subgroup analysis, most results did not change significantly. Sensitivity analysis demonstrated that the results of this meta-analysis were stable, and the trials causing asymmetry were mainly due to reporting bias.

### Possible explanation of the findings

Despite our findings that acupuncture is superior to sham acupuncture, some trials reported that sham acupuncture had similar effects to medication or acupuncture. Moreover, some heterogeneity still could not be explained by subgroup analysis. Thus, this paper will consider possible explanations.

#### Potential sources of heterogeneity

Owing to the aim of this research, which was to evaluate the efficacy of acupuncture for migraine, the possible acupuncture techniques should be considered. Specifically, different operators provided different stimulation parameters, which may result in different effects for participants. Additionally, different acupoints were selected for the included trials, which may also cause clinical heterogeneity. Considering the clinical acupuncture procedure, different stimulation parameters and acupoints may be potential sources of heterogeneity.

#### Low quality of evidence

Some studies have reported that there are few studies regarding acupuncture for migraine with a high quality of evidence (Zhang et al., 2008; Yang et al., 2012). Correspondingly, when selecting literature, 33 papers were excluded for data errors. Further, it is difficult to completely blind all participants and operators, especially for acupuncture operators. Blinding could only be applied for statistic staff and evaluators. Thus, blinding may have some influence on results. In our analysis, most of the included trials did not describe the details of the blinding, and some trials inappropriately designed the sham acupuncture procedure, such as the needle not piercing the skin. Inappropriate sham procedures obviously differ from acupuncture, so patients can easily see through the blinding, which will influence the results.

#### Lacking unified standards for sham acupuncture

There are no uniform standards for sham acupuncture, and disputes have arisen regarding sham acupuncture methods used in clinical research (Lund et al., 2009; Jiang et al., 2011; Zhou, 2017). Even some so-called sham acupuncture procedures may have the same effects as the acupuncture. According to current research, sham acupuncture using superficial insertion at acupoints, is also a common therapy, such as eye-acupuncture or ear-acupuncture. A previous study also indicated that even if there is no insertion, placebo needling could also stimulate the amyelinic afferent nerve, thereby affecting the pain transduction (Lund and Lundeberg, 2006). Therefore, superficial insertion cannot simply be regarded as a sham acupuncture. Further, in related trials in which verum and sham acupuncture had similar effects, it is not possible to determine that acupuncture only had a placebo effect. In terms of nonacupoint sham procedures, these are problematic in that the range of acupoint areas has not been defined or confirmed. Further, a study held that acupoints would exhibit sensitization and that the range of acupoints could be enlarged under disease (Qi et al., 2017). Thus, it cannot be determined if areas nearby the acupoints belong to nonacupoint areas.

### Quality of the evidence

The methodological quality of the included trials was variable. Methods for sequence generation, data handling for dropout or loss to follow-up and reporting were appropriate in most trials. However, ~1/3 of trials were at high risk for allocation concealment, and 1/3 were unclear without detailed allocation concealment. Furthermore, blinding was not adequately described in most studies. This analysis determined that the overall quality of the evidence for most outcomes was of low to moderate quality. Reasons for diminished quality consist of the following: no mentioned or inadequate allocation concealment, great probability of reporting bias, study heterogeneity, sub-standard sample size, and dropout without analysis.

### Strength and limitations

#### Agreements and disagreements with other studies or reviews

16 systematic reviews have evaluated the effects of acupuncture for migraine (Gao et al., 2011; Zheng and Cui, 2012; Chen, 2014; Yang et al., 2014, 2015; Zhao, 2014a, 2016; Dai and Lin, 2015; Jiang, 2015; Linde et al., 2016; Pu, 2016; Pu et al., 2016, 2017; Song et al., 2016; Xian, 2016; Chen et al., 2018) and the related details are available in Table 2. Results from those researches all showed that acupuncture had certain effects for migraine and were consistent with our meta-analysis. A recent Cochrane systematic review (Linde et al., 2016) reported that acupuncture had an effect for migraine over sham acupuncture, but this effect was small. Acupuncture might be at least similarly effective as treatment with prophylactic drugs. According to Dai et al. Dai and Lin (2015), the effective rate is high in acupuncture than in medication while the cure rates is similar between acupuncture and medication. However, unlike previous results, our analysis found that acupuncture is obviously better than sham acupuncture and medication.

**Table 2:** List and information of previous systematic reviews analyzing acupuncture therapy for migraine.

**Researches**	**Language**	**Type**	**No. of trials**	**Comparisons**	**Primary outcomes**	**Search fields**
Gao et al., 2011	Chinese	Migraine	12	AC vs. SA	Effective rates, days of migraine	PubMed, Cochrane Library, CBM
Linde et al., 2016	English	Episodic migraine	22	AC vs. NT, AC vs. SA, AC vs. ME	headache frequency at completion of treatment and at follow-up (closest to 6 months after randomization), response (at least 50% reduction in migraine), number of participants dropping out due to adverse effects	Cochrane Library, MEDLINE, EMBASE, AMED, ICTRP, Clinical Trials.gov, reference lists of all eligible studies
Song et al., 2016	Chinese	Migraine	18	AC/EA (or with placebo) vs. ME (or with placebo)	Shor-term effective rates (1–3 months), long-term effective rates (>3 months), days of migraine, adverse events	Cochrane Library, PubMed, Medline, CNKI, VIP, Wanfang data, Google website and Baidu website
Zheng and Cui, 2012	Chinese	Migraine	33	AC vs. ME, AC vs. TCM, AC vs. SA	Effective rates	PubMed, EMbase, CBM, CNKI, VIP, Wanfang data
Pu, 2016	Chinese	Acute migraine	5	AC vs. SA	VAS scores (2 h after treatment)	PubMed, Medline, Cochrane Library, CBM, CNKI, Wanfang data
Yang et al., 2014	Chinese	Migraine	10	AC vs. Flunarizine	Shor-term effective rates, long-term effective rates, migraine index, adverse events	PubMed, Cochrane Library, CNKI, VIP, Wanfang data
Zhao, 2016	Chinese	Menstrual migraine	30	AC vs. ME, AC vs. TCM, BT vs. TCM, AC with ear-acupuncture vs. ME	Total effective rates, migraine index, headache level, lasting time of migraine, adverse events	CBM, CNKI, VIP, CMCC, PubMed, EMBASE, SCI, Cochrane Library
Dai and Lin, 2015	Chinese	Migraine	2	AC vs. ME	Cure rates, Significantly effective rates, effective rates	PubMed, Cochrane Library, CNKI, VIP, Wanfang data
Xian, 2016	Chinese	Migraine prophylaxis	26	AC vs. NT, AC vs. SA, AC vs. ME	Effective rates, days of migraine, frequency of migraine, headache level, medication use, lasting time of migraine attack, PDI, MIDAS, SF-36, SF-12, adverse events	PubMed, Cochrane Library, EMBASE, CNKI, VIP, Wanfang data, CBM, TCM database
Jiang, 2015	Chinese	Migraine	18	EA vs. ME, EA with other therapy vs. ESA and EA vs. AC	Total effective rates, VAS scores	Medline, Cochrane Library, EMBASE, CNKI, VIP, Wanfang data, CBM
Chen, 2014	Chinese	Migraine	18	AC vs. ME, AC vs. Other Chinese therapy, AC vs. Other treatment	Effective rates, Migraine attack, lasting time of migraine, accompanying symptoms, TCD results, VAS scores, adverse events	Medline, Cochrane Library, EMBASE, CNKI, VIP, Wanfang data, CBM
Pu, 2016	Chinese	Migraine	7	AC vs. ME	Effective rates, days of migraine, migraine attack, medication use, rates of adverse events	Medline, Cochrane Library, EMBASE, CNKI, VIP, Wanfang data, CBM
Zhao, 2011	Chinese	Migraine	17	EA vs. ME, EA with other therapy vs. ME, EA vs. AC	Effective rates, VAS scores	PubMed, Cochrane Library, EMBASE, CNKI, VIP, CBM
Chen et al., 2018	Chinese	Migraine	18	AC vs. ME	Total effective rates, rates of adverse events, recurrence rate	CNKI, VIP, Wanfang data
Pu et al., 2017	Chinese	Migraine prophylaxis	7	AC vs. ME	Effective rates, days of migraine, VAS scores, rates of adverse events	PubMed, Cochrane Library, EMBASE, CNKI, VIP, Wanfang data, CBM
Yang et al., 2015	Chinese	Migraine	10	AC vs. SA	Overall response, headache characteristics, accompanying symptoms, medication use, adverse events	PubMed, Cochrane Library, Web of Science, CNKI, VIP, Wanfang data, CBM

*No, number; AC, acupuncture; SA, sham acupuncture; ME, medication; EA, electroacupuncture; SEA, sham electroacupuncture; TCM, traditional Chinese medicine; BT, bleeding therapy; NT, no treatment*.

Compared with previous researches, we focused on the effect of manual acupuncture, other than the broadly-defined acupuncture and excluded studies of acupoint injection, eye-acupuncture, etc. We found that 43.8% of systematic reviews only included one comparison; while in our research, the control group received no treatment, sham acupuncture or medication so that the range of comparisons could be more comprehensive. Besides, both search fields and time range we searched was broader than that in the previous meta-analysis. Moreover, we included more trials with the latest evidence than the previous researches. It could be that the range we searched and analyzed were more comprehensive than before. In addition, the outcomes that we measured were more comprehensive. All the previous meta-analysis included outcomes related to efficacy or safety of treatment. Specifically, our focuses on primary outcomes included not only efficacy and safety but also the evaluated patient-related outcomes, such as MSQ scores and SDS scores. Through MSQ scores and SDS scores, we could evaluate the psychological states and life quality after treatment. By assessing the efficacy of treatment and quality of life in migraine patients, we aimed to comprehensively verify acupuncture's improvement of the quality of life in migraine patients. Thus, this analysis could evaluate many aspects of the effectiveness of acupuncture for migraine, which is significantly different from previous systematic reviews. Furthermore, our study also considered the sources of heterogeneity and conducted subgroup analysis. Therefore, our research analyzed the included studies more systematically than previous researches and the results of this study are reliable.

#### Limitations of study

It is possible that some trials were published in other languages and were therefore not included in this analysis. Besides, it cannot rule out that some trials with small sample size have not been published. In future research, we would aim to identify these studies. Additionally, taken the vast difference in the number of acupoints, the theory of acupoint selection, and the operation, we only evaluated the overall efficacy and safety of acupuncture rather than the acupoint itself. The different acupoints and their combination may cause different effect, which deserves further exploration.

### Implications

#### Implications for practice

Acupuncture has certain efficacy both in the treatment and prevention of migraine and is superior to no treatment, sham acupuncture and medication. Additionally, acupuncture is safer than medication. Acupuncture may be an appropriate treatment for migraine, especially for patients who cannot tolerate preventive pharmacotherapy or the accompanying adverse events.

#### Implications for research

Based on the less rigorous design used in most acupuncture clinical trials, there is an urgent need to improve the quality of evidence in the design phase. First, appropriate randomized methods should be used, such as tables of random numbers and computer-controlled random sequence generation. Blinding should also be implemented for participants, statistics staff and assessors. Additionally, it may be better to separate participants into different groups, to avoid communication with each other. If sham acupuncture is used as a comparison, some well-designed placebo needles, such as the Streiberger needle, should be utilized (Xie et al., 2013). Further, sample size calculations should be made before trials.

For the population, none of the included studies reported the races of the populations, the researchers should specify the research objects that the results are applicable to. For outcomes, pain-related outcomes can directly reflect the relief of migraine, while outcomes like MSQ, SDS and SAS scores can show the efficacy on living quality and psychology etc. We suggest researchers use different outcomes to evaluate the efficacy for migraine instead of a single effective rate. To optimize the outcome evaluation, we are planning to establish a core outcome set for migraine. For research follow-up, trials with more than 6-months follow-up are rare. The current researches mainly focus on the short-term efficacy. More longitudinal studies evaluating the long-term effects of acupuncture for migraine are needed.

Moreover, the clinical trials on migraine lack uniform criteria regarding diagnosis, outcome selection, and reports. If both patients and doctors can be involved in setting these standards, it will be helpful for establishing standard criteria regarding migraine diagnosis, treatment and reports. In addition, it would be helpful to develop an optimal design of acupuncture that quantifies and regulates acupoints selection, manipulation, depth, angle, frequency, etc. Data from large studies with a high quality of evidence, such as clinical RCTs, may help doctors to select the most suitable treatment. Thus, further well-designed comparisons regarding acupuncture are needed.

## Conclusions

Acupuncture is superior to no treatment, sham acupuncture and medication, both in terms of efficacy and safety. Acupuncture could be recommended as one of the effective therapies for migraine. However, evidence are not enough to guide the operation of acupuncture for migraine, including the acupoints selection, the best course and frequency of treatment. Further large-sample, well-designed studies examining the effectiveness of acupuncture are needed.

## Author contributions

YJ, PB, and HC contributed equally to this paper. G-HT, Y-PL and YJ developed the research ideas and then designed the search strategy. G-HT, X-YT, X-LW, X-YL, and H-QC screened the abstract and full text. G-HT, X-YT, X-LW, X-YL, H-QC, and Y-YH extracted the data. X-YT, H-QC, and Y-YH evaluated the risks of bias. X-YT analyzed the data and is the guarantor. YJ, H-QC and X-YT wrote the first draft of the manuscript. PB and HC interpreted the results, refined the idea of the study. X-YZ revised the draft, including the language and managed the submission. All authors interpreted the data analysis and critically revised the manuscript.

### Conflict of interest statement

The authors declare that the research was conducted in the absence of any commercial or financial relationships that could be construed as a potential conflict of interest. The reviewer TS and handling Editor declared their shared affiliation.
