# Supplementary material for: The Effect of Acupuncture on the Quality of Life in Patients With Migraine: A Systematic Review and Meta-Analysis
Source: Front Pharmacol. 2018 Oct 26;9:1190. doi: 10.3389/fphar.2018.01190 (PMC6212461; doi:10.3389/fphar.2018.01190)
Supplement: Supplementary file 1 [file Data_Sheet_1.PDF]

Supplementary Material

The Effect of Acupuncture on the Quality of Life in Patients with Migraine: A Systematic Review and Meta-analysis

Xiao-Yi Tang[3#], Peng Bai[3#], Hao Chen[2#], He-Qing Chen[3], Ye-yin Hu[3], Xiao-li Wang[3], Xin-yi Li[3], You-Ping Li[1], Gui-hua Tian[1,3*]

Correspondence: Gui-hua Tian.: rosetgh@163.com

Contents

1 Supplementary PRISMA 2009 Checklist 2
2 Supplementary search terms and strategies 3
2.1 Searches for PubMed: 3
2.2 Searches for Web of Science: 3
2.3 Searches for Cochrane Library: 4
2.4 English version for Chinese search strategies: 5
3 Supplementary figures 6
4 Supplementary tables 12

1 Supplementary PRISMA 2009 Checklist

Section/topic	#	Checklist item	Reported on page #
**TITLE**			
Title	1	Identify the report as a systematic review, meta-analysis, or both.	1
**ABSTRACT**			
Structured summary	2	Provide a structured summary including, as applicable: background; objectives; data sources; study eligibility criteria, participants, and interventions; study appraisal and synthesis methods; results; limitations; conclusions and implications of key findings; systematic review registration number.	1
**INTRODUCTION**			
Rationale	3	Describe the rationale for the review in the context of what is already known.	2
Objectives	4	Provide an explicit statement of questions being addressed with reference to participants, interventions, comparisons, outcomes, and study design (PICOS).	2
**METHODS**			
Protocol and registration	5	Indicate if a review protocol exists, if and where it can be accessed (e.g., Web address), and, if available, provide registration information including registration number.	No protocol
Eligibility criteria	6	Specify study characteristics (e.g., PICOS, length of follow-up) and report characteristics (e.g., years considered, language, publication status) used as criteria for eligibility, giving rationale.	3-4
Information sources	7	Describe all information sources (e.g., databases with dates of coverage, contact with study authors to identify additional studies) in the search and date last searched.	3
Search	8	Present full electronic search strategy for at least one database, including any limits used, such that it could be repeated.	Supplementary Data
Study selection	9	State the process for selecting studies (i.e., screening, eligibility, included in systematic review, and, if applicable, included in the meta-analysis).	4-5
Data collection process	10	Describe method of data extraction from reports (e.g., piloted forms, independently, in duplicate) and any processes for obtaining and confirming data from investigators.	5
Data items	11	List and define all variables for which data were sought (e.g., PICOS, funding sources) and any assumptions and simplifications made.	5-6
Risk of bias in individual studies	12	Describe methods used for assessing risk of bias of individual studies (including specification of whether this was done at the study or outcome level), and how this information is to be used in any data synthesis.	5
Summary measures	13	State the principal summary measures (e.g., risk ratio, difference in means).	5

Synthesis of results	14	Describe the methods of handling data and combining results of studies, if done, including measures of consistency (e.g., I^2) for each meta-analysis.	5

2 Supplementary search terms and strategies

2.1 Searches for PubMed:

1. migraine.mh;
2. Migraine*.tiab;
3. status migrainosus*.tiab;
4. sick headache*.tiab;
5. 1 or 2 or 3 or 4;
6. acupuncture.mh;
7. acupuncture*.tiab;
8. needle*.tiab;
9. needling*.tiab;
10. thorns*.tiab;
11. dry-needling*.tiab;
12. body-acupuncture*.tiab;
13. stitch*.tiab;
14. tapping*.tiab;
15. electroacupuncture*.tiab;
16. electro-acupuncture*.tiab;
17. prick*.tiab;
18. pricking*.tiab;
19. bloodletting*.tiab;
20. puncturing collateral*.tiab;
21. bleeding therapy*.tiab;
22. Acusector*.tiab;
23. quick puncture*.tiab;
24. blood-letting*.tiab;
25. 6 or 7 or 8 or 9 or 10 or 11 or 12 or 13 or 14 or 15 or 16 or 17 or 18 or 19 or 20 or 21 or 22 or 23 or 24;
26. clinical trials as topic.mh;
27. clinical trials, phase I as topic.mh;
28. observational study as topic.mh;
29. 27 or 28;
30. 26 not 29;
31. clinical trials.pt;
32. clinical trials, phase I.pt;
33. observational study.pt;
34. 32 or 33;
35. 31 not 34;
36. random*;
37. 30 or 35 or 36;
38. 5 and 25 and 37.

2.2 Searches for Web of Science:

1. migraine*.mp;
2. status migrainosus*.mp;
3. sick headache*.mp;
4. 1 or 2 or 3;
5. acupuncture*.mp;
6. needle*.mp;
7. needling*.mp;
8. thorns*.mp;
9. dry-needling*.mp;
10. body-acupuncture*.mp;
11. stitch*.mp;
12. tapping*.mp;
13. electroacupuncture*.mp;
14. electro-acupuncture*.mp;
15. prick*.mp;
16. pricking*.mp;
17. bloodletting*.mp;
18. puncturing collateral*.mp;
19. bleeding therapy*.mp;
20. acusector*.mp;
21. quick puncture*.mp;
22. blood-letting*.mp;
23. 6 or 7 or 8 or 9 or 10 or 11 or 12 or 13 or 14 or 15 or 16 or 17 or 18 or 19 or 20 or 21 or 22
24. random*.mp;
25. 4 and 23 and 24.

2.3 Searches for Cochrane Library:

1. exp migraine. mh;
2. migraine*:ti,ab,kw;
3. status migrainosus*:ti,ab,kw;
4. sick headache*:ti,ab,kw;
5. 1 or 2 or 3 or 4;
6. exp acupuncture.mh;
7. acupuncture*:ti,ab,kw;
8. needle*:ti,ab,kw;
9. needling*:ti,ab,kw;
10. thorns*:ti,ab,kw;
11. dry-needling*:ti,ab,kw;
12. body-acupuncture*:ti,ab,kw;
13. stitch*:ti,ab,kw;
14. tapping*:ti,ab,kw;
15. electroacupuncture*:ti,ab,kw;
16. electro-acupuncture*:ti,ab,kw;
17. prick*:ti,ab,kw;
18. pricking*:ti,ab,kw;
19. bloodletting*:ti,ab,kw;

20. puncturing collateral*:ti,ab,kw;
21. bleeding therapy*:ti,ab,kw;
22. acusector*:ti,ab,kw;
23. quick puncture*:ti,ab,kw;
24. blood-letting*:ti,ab,kw;
25. 6 or 7 or 8 or 9 or 10 or 11 or 12 or 13 or 14 or 15 or 16 or 17 or 18 or 19 or 20 or 21 or 22 or 23 or 24,

2.4 English version for Chinese search strategies:

1. migraine.mp;
2. migraine.mp;
3. migraine due to wind syndrome.mp;
4. 1 or 2 or 3;
5. acupuncture and moxibustion.mp;
6. acupuncture.mp;
7. body-acupuncture.mp;
8. acupuncture therapy.mp;
9. thorns.mp;
1. needle.mp;
2. electroacupuncture.mp;
3. triangular needle.mp;
4. stinging.mp;
5. bleeding.mp;
6. pricking uperficial small vessels.mp;
7. pricking uperficial vessels.mp;
8. stitch.mp;
9. percussopunctator.mp;
10. seven-star needle.mp;
11. lohan needle.mp;
12. dermal needle.mp;
13. tapping.mp;
14. three part of needling for head.mp;
15. 5 or 6 or 7 or 8 or 9 or 10 or 11 or 12 or 13 or 14 or 15 or 16 or 17 or 18 or 19 or 20 or 21 or 22 or 23;
16. random.mp;
17. 4 and 24 and 25.

3 Supplementary figures

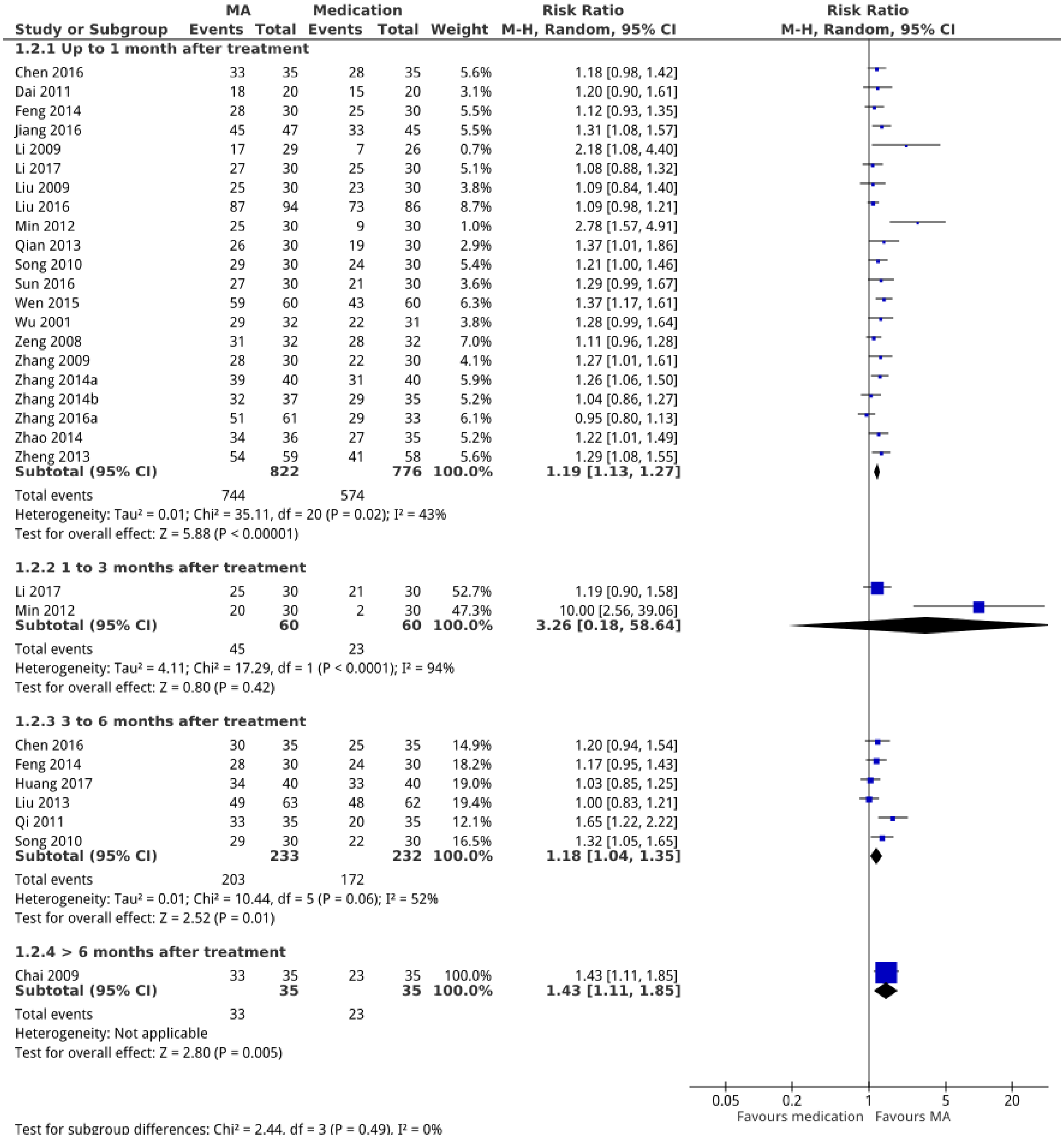

Figure a. Forest plot-acupuncture vs. medication-total effective rate Abbreviations: MA, manual acupuncture.

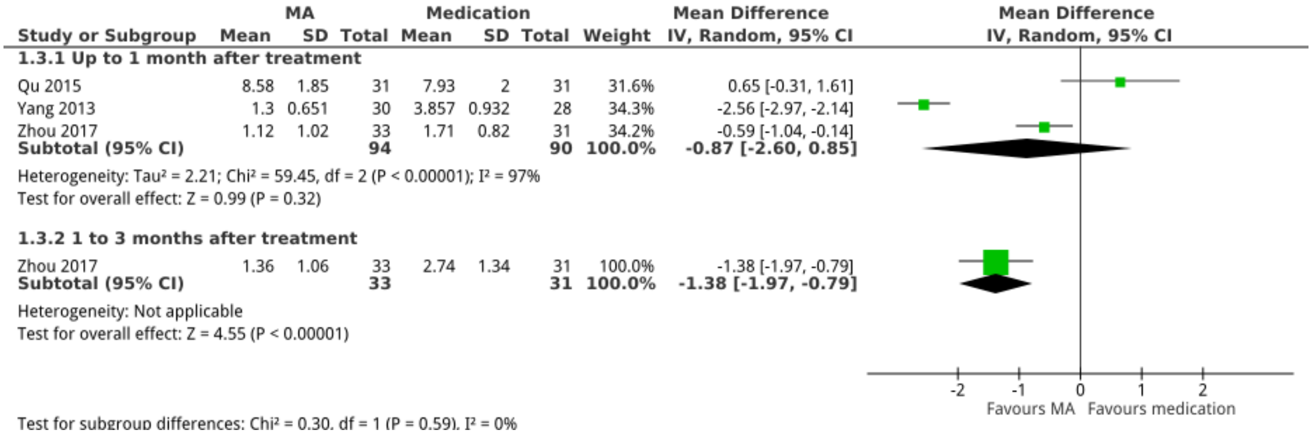

Figure b. Forest plot-acupuncture vs. medication-days of attack Abbreviations: MA, manual acupuncture.

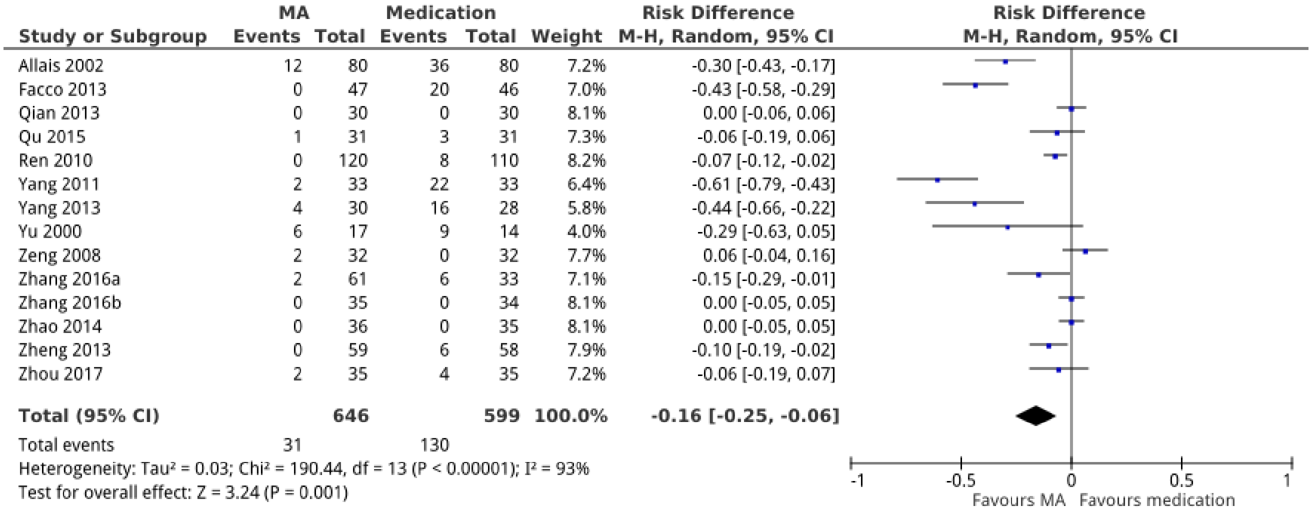

Figure c. Forest plot-acupuncture vs. medication-adverse events Abbreviations: MA, manual acupuncture.

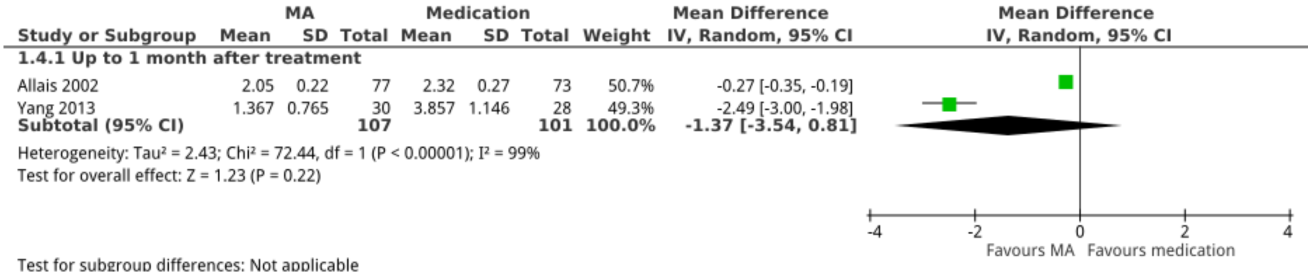

Figure d. Forest plot-acupuncture vs. medication-attack frequency Abbreviations: MA, manual acupuncture.

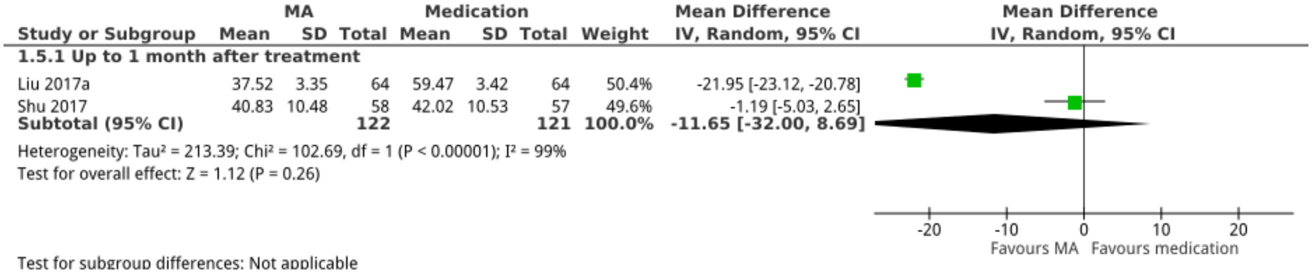

Figure e. Forest plot-acupuncture vs. medication-SDS scores Abbreviations: MA, manual acupuncture; SDS, Self-rating Depression Scale.

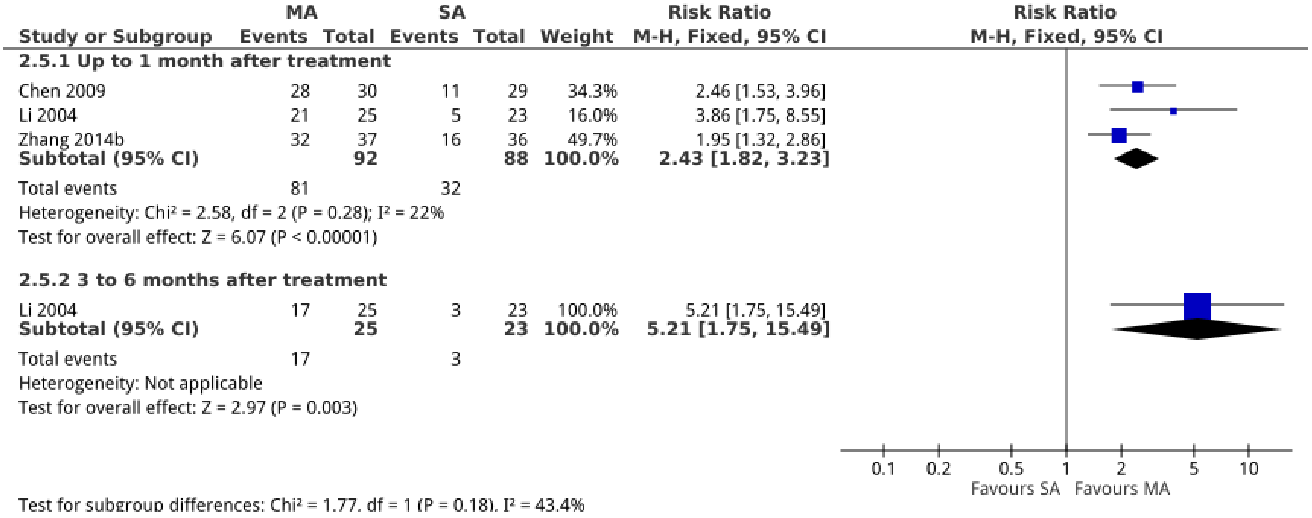

Figure f. Forest plot-acupuncture vs. sham acupuncture-total effective rate

Abbreviations: MA, manual acupuncture; SA, sham acupuncture.

	MA			SA				Mean Difference
Study or Subgroup	Mean	SD	Total	Mean	SD	Total	Weight	IV, Random, 95% CI
**2.6.1 Up to 1 month after treatment**								
Alecrim 2005	4.8	3.6	32	7.3	5	32	13.5%	-2.50 [-4.63, -0.37]
Alecrim 2006	6.4	3.3	14	6.3	3.1	14	12.1%	0.10 [-2.27, 2.47]
Alecrim 2008	4.8	3	18	7.3	5	32	13.0%	-2.50 [-4.72, -0.28]
Linde 2005	4.9	3.4	145	4.7	3.4	81	21.7%	0.20 [-0.72, 1.12]
Wallasch 2012	4.13	2.55	13	4.82	1.98	14	16.0%	-0.69 [-2.42, 1.04]
Wang 2015	5.2	5	26	10.1	7.1	24	7.7%	-4.90 [-8.33, -1.47]
Zhao 2011	3.8	2.56	32	5.09	2.55	11	15.9%	-1.29 [-3.04, 0.46]
**Subtotal (95% CI)**			**280**			**208**	**100.0%**	**-1.30 [-2.45, -0.16]**
Heterogeneity: Tau² = 1.35; Chi² = 15.52, df = 6 (P = 0.02); I² = 61%								
Test for overall effect: Z = 2.23 (P = 0.03)								
**2.6.2 1 to 3 months after treatment**								
Alecrim 2005	5.2	3.6	32	6.5	4.8	32	21.8%	-1.30 [-3.38, 0.78]
Alecrim 2006	6.2	4.3	13	4.2	4.8	13	15.7%	2.00 [-1.50, 5.50]
Linde 2004	5.3	3.2	15	3.4	1.9	13	22.5%	1.90 [-0.02, 3.82]
Wallasch 2012	3.76	3.03	13	4.34	2.63	14	21.5%	-0.58 [-2.73, 1.57]
Wang 2015	5.1	3.7	26	9.5	6.2	24	18.4%	-4.40 [-7.26, -1.54]
**Subtotal (95% CI)**			**99**			**96**	**100.0%**	**-0.47 [-2.59, 1.64]**
Heterogeneity: Tau² = 4.23; Chi² = 15.55, df = 4 (P = 0.004); I² = 74%								
Test for overall effect: Z = 0.44 (P = 0.66)								
**2.6.3 3 to 6 months after treatment**								
Alecrim 2005	5.6	3.8	32	6.2	4.8	32	30.5%	-0.60 [-2.72, 1.52]
Alecrim 2006	4.4	2.8	12	4	2.3	12	32.6%	0.40 [-1.65, 2.45]
Alecrim 2008	7.4	4.4	17	6.2	4.5	17	15.3%	1.20 [-1.79, 4.19]
Linde 2004	5.8	4.6	15	4.1	1.8	13	21.5%	1.70 [-0.83, 4.23]
**Subtotal (95% CI)**			**76**			**74**	**100.0%**	**0.50 [-0.67, 1.67]**
Heterogeneity: Tau² = 0.00; Chi² = 2.12, df = 3 (P = 0.55); I² = 0%								
Test for overall effect: Z = 0.83 (P = 0.41)								
**2.6.4 > 6 months after treatment**								
Linde 2004	6	4.1	15	4.1	2.9	13	71.4%	1.90 [-0.71, 4.51]
Wang 2015	11.3	5.5	16	12.3	5.6	9	28.6%	-1.00 [-5.54, 3.54]
**Subtotal (95% CI)**			**31**			**22**	**100.0%**	**1.07 [-1.50, 3.64]**
Heterogeneity: Tau² = 0.63; Chi² = 1.18, df = 1 (P = 0.28); I² = 15%								
Test for overall effect: Z = 0.82 (P = 0.41)								

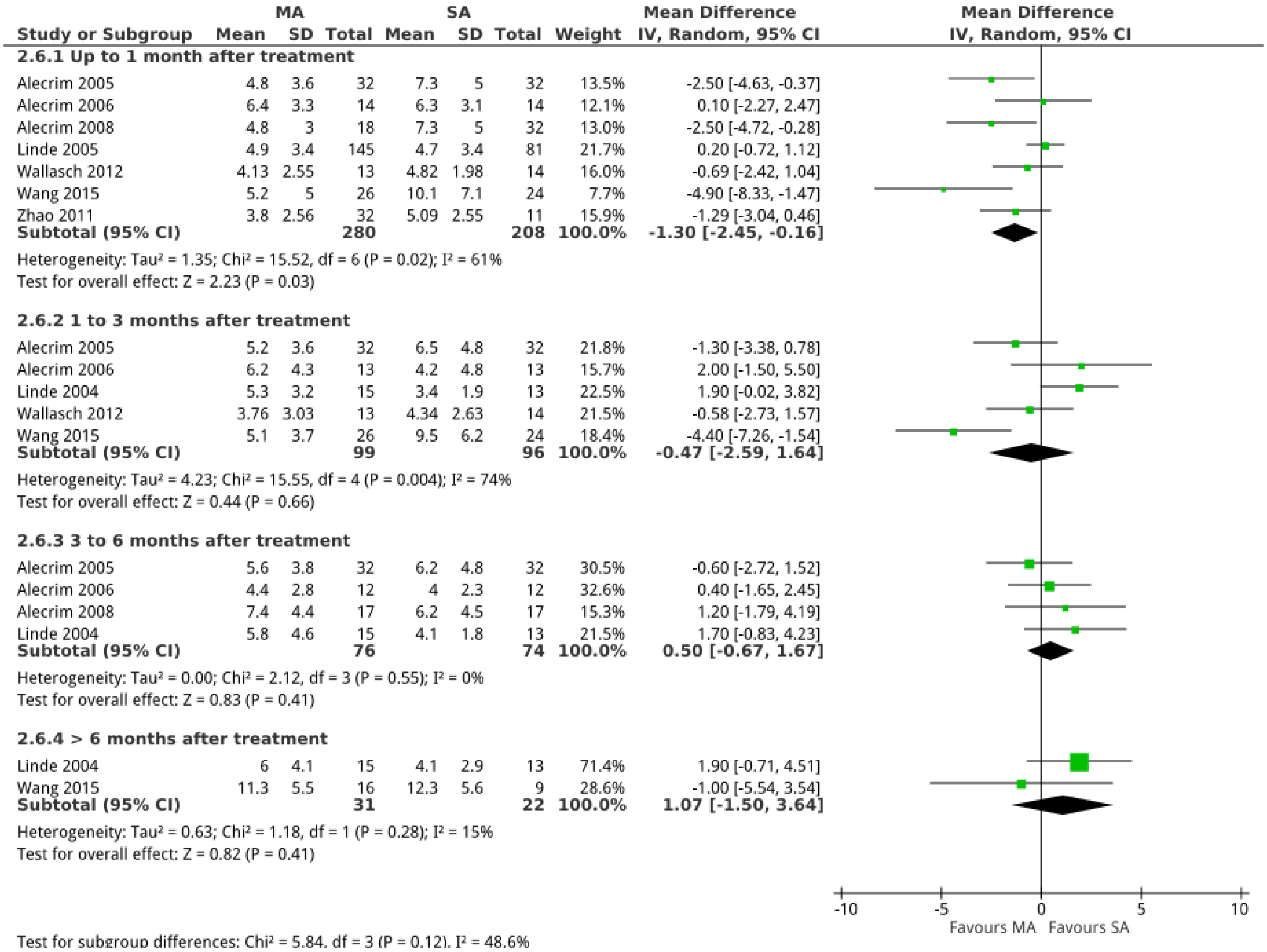

Test for subgroup differences: Chi² = 5.84, df = 3 (P = 0.12), I² = 48.6%

Figure g. Forest plot-acupuncture vs. sham acupuncture-days of attack Abbreviations: MA, manual acupuncture; SA, sham acupuncture.

Study or Subgroup	MA Mean	MA SD	MA Total	SA Mean	SA SD	SA Total	Weight	Mean Difference IV, Random, 95% CI
**2.7.1 Up to 1 month after treatment**								
Alecrim 2005	2.5	1.5	32	3.2	1.3	32	13.4%	-0.70 [-1.39, -0.01]
Alecrim 2006	3.5	1.5	14	3.8	1.7	13	8.5%	-0.30 [-1.51, 0.91]
Alecrim 2008	3.1	1.5	18	3.2	1.5	17	10.3%	-0.10 [-1.09, 0.89]
Jiang 2011	1.5	1.434	10	2.545	2.423	11	5.7%	-1.04 [-2.73, 0.64]
Linde 2004	2.5	1	15	1.9	0.95	13	13.0%	0.60 [-0.12, 1.32]
Linde 2005	1.5	1.2	145	1.6	1.3	81	16.8%	-0.10 [-0.44, 0.24]
Marcus 2008	1	1.2	9	1.8	1.7	10	7.8%	-0.80 [-2.11, 0.51]
Mohsen 2014	3	0.9	50	4.2	1.1	50	16.4%	-1.20 [-1.59, -0.81]
Zhao 2011	3	1.52	32	4.18	1.94	11	8.1%	-1.18 [-2.44, 0.08]
**Subtotal (95% CI)**			**325**			**238**	**100.0%**	**-0.48 [-0.96, -0.00]**
Heterogeneity: Tau² = 0.33; Chi² = 28.97, df = 8 (P = 0.0003); I² = 72%								
Test for overall effect: Z = 1.96 (P = 0.05)								
**2.7.2 1 to 3 months after treatment**								
Alecrim 2005	2.7	1.5	32	3.2	1.3	32	20.1%	-0.50 [-1.19, 0.19]
Alecrim 2006	3.4	1.3	13	2.4	1.4	13	19.8%	1.00 [-0.04, 2.04]
Linde 2004	2.3	1.7	15	2.1	0.8	13	19.9%	0.20 [-0.76, 1.16]
Mohsen 2014	4.2	1	50	4.8	1	50	20.3%	-0.60 [-0.99, -0.21]
Shai 1997	1.4	0.6	12	9.3	1.4	10	19.9%	-7.90 [-8.83, -6.97]
**Subtotal (95% CI)**			**122**			**118**	**100.0%**	**-1.56 [-4.07, 0.96]**
Heterogeneity: Tau² = 8.05; Chi² = 236.47, df = 4 (P < 0.00001); I² = 98%								
Test for overall effect: Z = 1.21 (P = 0.22)								
**2.7.3 3 to 6 months after treatment**								
Alecrim 2005	2.8	1.4	32	2.8	1.4	32	34.8%	0.00 [-0.69, 0.69]
Alecrim 2006	2.8	1.9	12	2.9	1.4	12	9.2%	-0.10 [-1.44, 1.24]
Alecrim 2008	3.7	0.9	17	3.2	0.9	17	44.8%	0.50 [-0.11, 1.11]
Linde 2004	2.4	2.3	15	1.6	0.6	13	11.2%	0.80 [-0.41, 2.01]
**Subtotal (95% CI)**			**76**			**74**	**100.0%**	**0.30 [-0.10, 0.71]**
Heterogeneity: Tau² = 0.00; Chi² = 2.16, df = 3 (P = 0.54); I² = 0%								
Test for overall effect: Z = 1.47 (P = 0.14)								

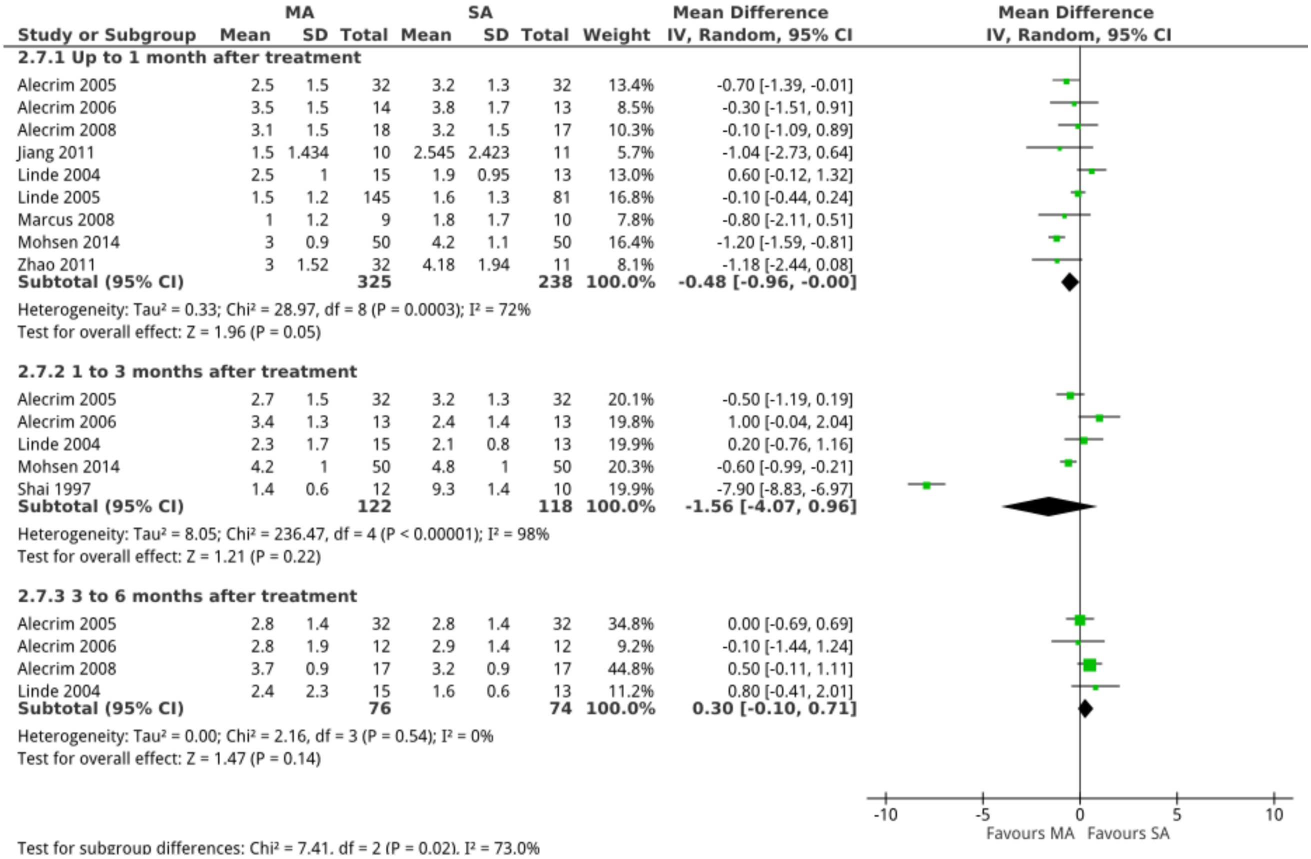

Test for subgroup differences: Chi² = 7.41, df = 2 (P = 0.02), I² = 73.0%

Figure h. Forest plot-acupuncture vs. sham acupuncture-attack frequency Abbreviations: MA, manual acupuncture; SA, sham acupuncture.

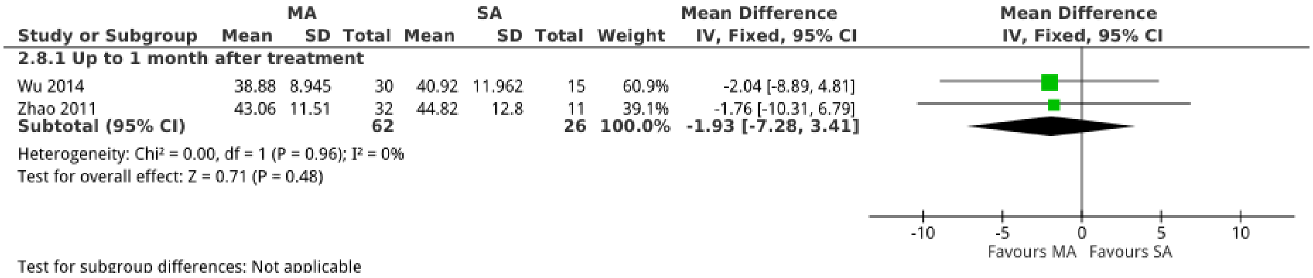

Figure i. Forest plot-acupuncture vs. sham acupuncture-SDS scores Abbreviations: MA, manual acupuncture; SA, sham acupuncture; SDS, Self-rating Depression Scale.

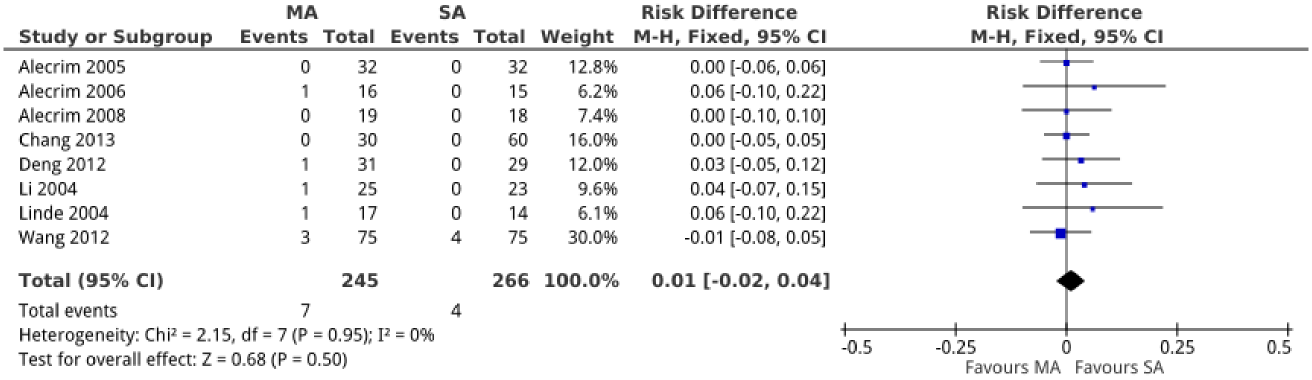

Figure j. Forest plot-acupuncture vs. sham acupuncture-adverse events Abbreviations: MA, manual acupuncture; SA, sham acupuncture

4 Supplementary tables

Supplementary Table 1-(1) Characteristics of studies included in systematic review.

Study	Diagnosis	Country	Mean age (T/C)	Gender (M:F) T/C	Included participates (T:C) / Analysis participates (T:C)
Alecrim,2005	migraine without aura	Brazil	Unknown	Unknown	64:64
Alecrim,2006	migraine without aura	Brazil	32.5/39.1	7:24	31/28
Alecrim,2008	migraine without aura	Brazil	35	4:33	37/36
Allais,2002	migraine	Italy	38.4±9.7/37.2±9.3	all female	80:80/77:73
Chai,2009	migraine	China	32.15/31	18:17/20:15	35:35/35:35
Chang,2013	migraine	China	38.89±12.8/38.66±11.43,42.58±11.98	13:17/10:20/19:11	30:60/30:60
Chen,2009	migraine	China	27.83±7.57/26.86±6.66	8:22/12:17	30:29/20:29
Chen,2016	migraine with liver-yang hyperactivity syndrome	China	46.78±10.26/45.61±9.88	16:19/15:20	35:35/35:35
Dai,2011	migraine	China	24-55/25-54	3:17/2:18	20:20/20:20

Study	Diagnosis	Country	Mean age (T/C)	Gender (M:F) T/C	Included participates (T:C) / Analysis participates (T:C)
Deng,2012	migraine without aura	China	32.26±11.72/30.43±8.06	4:27/6:23	31:29/31:29
Facco,2013	migraine without aura	Italy	40/34	14:27/14:27	50:50/41:41
Feng,2014	migraine with with syndrome of upward disturbance of phlegm-turbidity	China	15-65	20:40	30:30/30:30
Gao,2012	migraine without aura	China	18-45	3:12/3:12	16:16/15:15
Huang,2017	migraine	China	34.7±7.5/35.4±8.7	19:21/16:24	40:40/40:40
Jiang,2011	migraine without aura	China	31.6±12.703/26.0±6.957	2:8/1:10	12:12/10:11
Jiang,2016	migraine	China	38±11/39±9	12:35/9:36	47:45/47:45
Li,2004	migraine	China	32.08±6.92/31.78±7.56	8:17/7:16	25:23/25:23
Li,2009	migraine with liver-yang hyperactivity syndrome	China	Unknown	Unknown	29:26/29:26
Li,2010	migraine	China	35.3±9.8/36.7±9.2	17:54/18:53	71:71/71:71
Li,2017	migraine	China	43.87±9.59/43.33±8.65	13:17/14:16	30:30/30:30

Study	Diagnosis	Country	Mean age (T/C)	Gender (M:F) T/C	Included participates (T:C) / Analysis participates (T:C)
Liang,2016	chronic migraine	China	46.5/44.5	11:19/10:20	30:30/30:30
Linde ,2004	Menstrual migraine	Sweden	36	all female	31/28
Linde,2005	migraine	Germany	43	90:212	145:81:76/145:81:76
Liu,2009	Menstrual migraine	China	26.4±1.71/25.3±2.01	all female	30:30/30:30
Liu,2013	Menstrual migraine	China	25.34±6.21	all female	66:66/63:62
Liu,2016	chronic migraine	China	41.74±5.32/40.20±5.84	47:47/42:44	100:100/94:86
Liu,2017a	migraine	China	37.35±3.26/36.56±3.74	23:41/25:39	64:64/64:64
Liu,2017b	migraine without aura	China	38.21±22.23/35.43±21.54/37.76±22.11	41:19/22:8	60:30/60:30
Marcus,2008	migraine	Germany	43.5±8.5	2:17	17:13/12:11
Min,2012	migraine	China	32.23/32.40	11:19/13:17	30:30/30:30
Mohsen,2014	migraine without aura	Iran	35.8±10.9/37.2±11.2	21:29/20:30	50:50/the numbers of dropout is Unknown

Study	Diagnosis	Country	Mean age (T/C)	Gender (M:F) T/C	Included participates (T:C) / Analysis participates (T:C)
Qi,2011	migraine without aura	China	40.68/42.3	8:27/7:28	35:35/35:35
Qian,2013	Acute migraine	China	34.1±9.2/34.9±10.3	10:20/12:18	30:30/30:30
Qu,2015	migraine	China	34.2±8.9/33.6±6.4	14:17/16:15	31:31/31:31
Ren,2010	migraine with aura	China	19-78/18-77	49:71/45:65	120:110/120:110
Shai,1997	migraine for children	Israel	9.8±1.2/10.4±1.6	5:7/4:6	12:10/12:10
Shu,2017	migraine	China	47±9/47±8	26:34/23:37	60:60/58:57
Song,2010	migraine	China	34.2±7.59/ 34.8±7.60	8:22/10:20	30:30/30:30
Su,2016	migraine	China	36.70±9.32/35.93±8.65	16:19/15:17	35:32/35:32
Sun,2016	migraine without aura	China	18-45	21:39	30:30/30:30
Wallasch,2012	migraine	Germany	37.2±9.6/39.3±11.7	2:16/2:15	18:17/13:14
Wan,2013	migraine without aura, but with liver-yang hyperactivity syndrome	China	28:29	5:9/3:11	14:14/14:14

Study	Diagnosis	Country	Mean age (T/C)	Gender (M:F) T/C	Included participates (T:C) / Analysis participates (T:C)
Wang,2012	migraine	China	37.8±10.6/38.6±12.6	8:67/12:63	75:75/72:68
Wang,2015	frequent migraine	Australia	41.6 (14.9)/43.8 (13.4)	18:8/19:5	26:24/25:23
Wen,2015	Acute migraine	China	48.3±2.3/48.4±2.1	all female	60:60/60:60
Wu,2001	migraine	China	36.3±8.6	10:22/8:23	32:31/32:31
Wu,2014	migraine without aura	China	21.20±2.426/21.27±2.374/22.13	7:23/3:12	32:16/30:15
Yang,2011	migraine	China	47.6±7.4/48.1±6.4	3:30/4:29	33:33/32:32
Yang,2013	migraine	China	40.4±12.4/39.7±13.31	16:14/11:17	30:28/30:28
Yu,2000	Acute migraine	China	29.6/28.8	3:14/3:11	17:14/17:14
Zeng,2008	migraine without aura	China	42/41.875	9:23/11:21	33:33/32:32
Zeng,2015	migraine	China	39.89±12.78/40.13±12.27	18:16/17:17	34:34/34:34
Zhang,2009	migraine	China	32.97±14.68/36.63±6.32	10:20/11:19	30:30/30:30

Study	Diagnosis	Country	Mean age (T/C)	Gender (M:F) T/C	Included participates (T:C) / Analysis participates (T:C)
Zhang,2014a	migraine	China	35.5/33.25	19:21/17:23	40:40/40:40
Zhang,2014b	migraine	China	36.32±4.71/38.06±5.90/36.83±6.12	17:20/17:21/22:16	37:38:38/37:36:35
Zhang,2016a	migraine with syndrome of blood stasis blocking brain	China	43.89±10.73/43.22±12.05/41.63±11.13	19:42/10:23	61:33/60:33
Zhang,2016b	Vestibular migraine	China	41.24±10.25/42.13±10.24	16/19,14/20	36:36/35:34
Zhao,2011	migraine without aura	China	31.60±12.70/36.73±11.79/33.00±8.68/26.00±6.96	2:8/3:8/2:9/1:10	12:12:12:12/10:11:11:11
Zhao,2014	migraine without aura	China	37.64±11.00/36.77±10.98	9:27/10:25	36:36/36:35
Zheng,2013	migraine	China	34.54±6.71/35.62±5.05	21:38/19:39	60:60/59:58
Zheng,2016	chronic migraine	China	38.63±9.81/39.00±10.67	12:18/15:15	30:30/30:30
Zhou,2017	chronic migraine	China	38.4±10.3/40.2±11.8	10:25/11:24	35:35/33:31

Note: Abbreviations: T, intervention group; C, comparison group.

Supplementary Table 1-(2) Characteristics of studies included in systematic review.

Study	Intervention	Acupoints	Frequency	Duration of treatment	Comparison	Single dose / Acupoints	Frequency	Duration of treatment
Alecrim,2005	acupuncture	Unknown	2/w for the first 4w, 1/w for the next 8w	12w	Sham acupuncture	Superficial insertion at acupoints	2/w for the first 4w, 1/w for the next 8w	12w
Alecrim,2006	acupuncture	GB12/20/21, BL10	2/w for the first 4w, 1/w for the next 8w	12w	Sham acupuncture	Superficial insertion at acupoints	2/w for the first 4w, 1/w for the next 8w	12w
Alecrim,2008	acupuncture	GB12/20/21, BL10	2/w for the first 4w, 1/w for the next 8w	12w	Sham acupuncture	Superficial insertion at acupoints	2/w for the first 4w, 1/w for the next 8w	12w
Allais,2002	acupuncture	LR3, SP6, ST36, CV12, LI4, PC6, GB20, GB14, EX-HN5, GV20	1/w for first 2m, 1/m for the next 4m	6m	Flunarizine	10mg	1/d for first 2m, 1/d and 20d for the next 4m	6m
Chai,2009	acupuncture	SJ23, GB8, GB20, LI4, GV20, GB4, LU7, GV29, LR3	1/d	4w	Rotundine	60mg	3/d	4w

Study	Intervention	Acupoints	Frequency	Duration of treatment	Comparison	Single dose / Acupoints	Frequency	Duration of treatment
Chang,2013	acupuncture	SJ20, GB20, SJ5, GB34, GB40	Immediate Effect		Sham acupuncture	Deep insertion at nonacupoints	Immediate Effect	
Chen,2009	acupuncture	SJ20, SJ5, GB34, GB40	1/d	8w	Sham acupuncture	Deep insertion at nonacupoints	1/d	8w
Chen,2016	acupuncture	Ashi points, SJ5, GB41, LU7, GB20	1/d	2w	Flunarizine Hydrochloride	10mg	qn	2w
Dai,2011	acupuncture	GV20, LI4, LR3	qod	40d	Flunarizine Hydrochloride	10mg	qn	40d
Deng,2012	acupuncture	GB20, LI4, GB34, GB40	Immediate Effect		Sham acupuncture	Deep insertion at nonacupoints	Immediate Effect	
Facco,2013	acupuncture	GB8, BL12, ST8, GB20, BL60	2/w	10d a period, 2peirods	Valproic acid	300-600mg	1/d	3m
Feng,2014	acupuncture	PC6, GV26, SP6, GB8, LI11, CV12, SP9, ST43	1/d	4w	Flunarizine Hydrochloride	5mg	2/d	4w
Gao,2012	acupuncture	SJ5, GB34, GB40	Unknown	4w	Sham acupuncture	Deep insertion at nonacupoints	Unknown	4w
Huang,2017	acupuncture	GB20, EX-HN5, SJ20, GB8, LI4,	1/d	6m	Gabapentin	100mg	3/d	6m

Study	Intervention	Acupoints	Frequency	Duration of treatment	Comparison	Single dose / Acupoints	Frequency	Duration of treatment
		GB41, LR2						
Jiang,2011	acupuncture	SJ20, GB20, SJ5, GB34, GB40	4/w	16times	Sham acupuncture	Deep insertion at nonacupoints	4/w	16times
Jiang,2016	acupuncture	GV20, GV29, ST8, GB14, EX-HN5, GB8, SJ5, GB41, LR3, KI3	1/d	36d	Flunarizine Hydrochloride	10mg	qn	36d
Li,2004	acupuncture	GB20, EX-HN5, SJ5, LI4, GB41	1/d	10d a period, 1peirod	Sham acupuncture	GB20, EX-HN5, SJ5, LI4, GB41	1/d	10d a period, 1peirod
Li,2009	acupuncture	Ashi points, ST8, GB8, GB20, GB43, LR3	qod	2w	Flunarizine Hydrochloride	10mg	qn	2w
Li,2010	acupuncture	Ashi points	Immediate Effect		Ergot caffeine	2tablets	Immediate Effect	
Li,2017	acupuncture	GV20, GV29,EX-HN5, SJ23, GB8, GB20, GB34, LI4, LR3, KI3	3/w	4w	Aspirin	50mg	1/d	4w
Liang,2016	acupuncture	GB34, SJ20, GB40, SJ5	1/d	4w	Sham acupuncture	Deep insertion at nonacupoints	1/d	4w

Study	Intervention	Acupoints	Frequency	Duration of treatment	Comparison	Single dose / Acupoints	Frequency	Duration of treatment
Linde ,2004	acupuncture	GB8, GB20, LI4, LR3, SP6	8th,5th,3th days before the due menstrual date	3m	Sham acupuncture	Superficial insertion at nonacupoints	8th,5th,3th days before the due menstrual date	3m
Linde,2005	acupuncture	GB20, GB40/GB41/GB42, GV20, LR3, SJ3/SJ5	2/w for the first 4w, 1/w for the next 4w	8w	Sham acupuncture	Superficial insertion at nonacupoints	2/w for the first 4w, 1/w for the next 4w	8w
					Waiting-list group	none	none	
Liu,2009	acupuncture	LR3, LI4, ST8, EX-HN5, GB8, Ashi points	1/d	1w before menstruation as a period, 4periods	Aspirin	0.3g-0.6g	3/d	7d as a period when it attacks, 4 periods
Liu,2013	acupuncture	GB20, GB8, GV20	3/w	3times a period, 3periods	Flunarizine Hydrochloride	10mg	1/d	1w a period, 3periods
Liu,2016	acupuncture	GV20, GV23, GB8, GB20, LI4, LR3	1/d	10d a period, 4peirods	Compound paracetamol tablets, Flunarizine Hydrochloride, Vitamin, Methylcobalamin	10mg, 5mg, 10mg, 0.5mg	3/d, qn, 3/d, 3/d	10d a period, 4peirods
Liu,2017a	acupuncture	EX-HN5, GV20, LI4, ST36, SJ3, ST8, SJ10, LR3, GB44, PC6, CV12, GB20,	1/d or 2/d if severe	2w	Carbamazepine	0.1g	3/d	21d

Study	Intervention	Acupoints	Frequency	Duration of treatment	Comparison	Single dose / Acupoints	Frequency	Duration of treatment
		GB17						
Liu,2017b	acupuncture	GB20, EX-HN5, ST8, GB8, Ashi points	1/d	10d a period, 2peirods	Flunarizine Hydrochloride	5mg	1/d	10d a period, 2peirods
Marcus,2008	acupuncture	GB41, LR3, GB20, GV20, EX-HN5	Unknown	12sessions	Sham acupuncture	Superficial insertion at nonacupoints	unknown	12sessions
Min,2012	acupuncture	GB20, GV20, GB8, GB7, SJ23, GB15, GB43, GB41, SJ3, Sj5	1/d	3w	Flunarizine Hydrochloride	5mg	1/d	3w
Mohsen,2014	acupuncture	Unknown	3/w	4w	Sham acupuncture	Superficial insertion at nonacupoints	3/w	4w
Qi,2011	acupuncture	EX-HN5, GB5, GB8, SJ20, SJ5, PC6, GB20, LR3, GB41, GB7, ST8, SJ4, PC3, GV16	2/w	3m	Flunarizine Hydrochloride	10mg	qn	3m
Qian,2013	acupuncture	ST8, GB8, GB20, SJ5, GB34, LR3	1/d	28d	Flunarizine Hydrochloride, Ibuprofen	5mg, 0.3g	1/d, 2/d	28d
Qu,2015	acupuncture	GB8, ST8, EX-HN5, GB20	5/w	4w	Flunarizine Hydrochloride	5-10mg	qn	4w

Study	Intervention	Acupoints	Frequency	Duration of treatment	Comparison	Single dose / Acupoints	Frequency	Duration of treatment
Ren,2010	acupuncture	EX-HN5, GB8, GB20, SJ17, GB41, SJ5	1/d	10d a period, 2peirods	Flunarizine Hydrochloride	5mg	qn	10d a period, 2peirods
Shai,1997	acupuncture	Unknown	1/w	10w	Sham acupuncture	Superficial insertion at acupoints	1/w	10w
Shu,2017	acupuncture	GV20, GV26, SP6, EX-HN5, GB8, GB20	3/w	4w	Flunarizine Hydrochloride	10mg	qn	4w
Song,2010	acupuncture	GB20, GV20, EX-HN5, EX-HN3, SJ5, HT7, LI4, KI3	1/d	4w	Flunarizine Hydrochloride	5mg	1/d	4w
Su,2016	acupuncture	PC6, GV20, GV26, GV29, LI4, LR3	1/d	30d	Flunarizine Hydrochloride	5-10mg	qn	30d
Sun,2016	acupuncture	GV20, GB20, EX-HN5, LI4, LR3	1/d	4w	Acute: Dihydroergotamine mesylate; Chronic:Propranolol	Acute: 2mg-6mg, Chronic: 10-60mg	Acute: 1/d; Chronic: 2/d	4w
Wallasch,2012	acupuncture	LI4, ST36, SJ5, GB41, SI3, BL62, GV20, GB20, EX-HN5, SJ23, LR3, KI3	1/w	8w	Sham acupuncture	Deep insertion at nonacupoints	1/w	8w
Wan,2013	acupuncture	SJ20, SJ5, GB34,	1/d	5times a period,	Sham acupuncture	Deep insertion at	1/d	5times a period,

Study	Intervention	Acupoints	Frequency	Duration of treatment	Comparison	Single dose / Acupoints	Frequency	Duration of treatment
		GB40		2periods		nonacupoints		2periods
Wang,2012	acupuncture	GV20, GV24, ST8, GB20	Immediate effects		Sham acupuncture	Deep insertion at nonacupoints	Immediate effects	
Wang,2015	acupuncture	GB20, EX-HN5, GB8, LI4	2/w for the first 4w, 1/w for the next 4w,1/2w for 9-12w and 1/m for last 2m	20w	Sham acupuncture	Superficial insertion at nonacupoints	2/w for the first 4w, 1/w for the next 4w,1/2w for 9-12w and 1/m for last 2m	20w
Wen,2015	acupuncture	GB20, EX-HN3, Niesanzhen, GB8, ST8, EX-HN5, GB20	3/w for first month, 2/w for next 2months	3m	Honglongzhentong Pill	3tablets	2/d	3m
Wu,2001	acupuncture	GB20, GB14, GB8, EX-HN5, SJ5, SJ3, GB41, GB10	1/d	10d a period, 1peirod	Indometacin	25mg	2/d	10d a period, 1peirod
Wu,2014	acupuncture	GB40, SJ5, GB34, LI6, ST42， ST36	1/d	5times a period, 4periods	Sham acupuncture	Deep insertion at nonacupoints	1/d	5times a period, 4periods
Yang,2011	acupuncture	BL2, GB20, EX-HN5, GB29	2/w	12w	Topiramate	25-100mg	1/d	12w

Study	Intervention	Acupoints	Frequency	Duration of treatment	Comparison	Single dose / Acupoints	Frequency	Duration of treatment
Yang,2013	acupuncture	SJ23, GB8, LI4, GB41	1/d	10d a period, 2peirods	Flunarizine Hydrochloride	5mg	unknown	30d
Yu,2000	acupuncture	LI4, SI3, SJ3	Immediate Effect		Sumatriptan	100mg	Immediate Effect	
Zeng,2008	acupuncture	GB13, GV16, GV23	1/d	5times a period, 4periods	Naproxen	0.25g	3/d	5times a period, 4periods
Zeng,2015	acupuncture	ST8, ST44, LI4, LU9, LIV3, SP6, GB1,GB14, GB20, GV20, GB29	1/d	7d a period, 3periods	Flunarizine Hydrochloride	5mg	1/10d	7d a period, 3periods
Zhang,2009	acupuncture	GB1, SJ23, GB8, SJ20, GB19, SJ19	1/d	7d a period, 2periods	Ergot caffeine; Flunarizine Hydrochloride	2tablets; 5mg	2/d; qn	7d a period, 2periods
Zhang,2014a	acupuncture	Ashi points, GB20, LR3	1/d	15d	Flunarizine Hydrochloride	10mg	2/d	15d
Zhang,2014b	acupuncture	GB20, SJ23, SJ5, GB41, Sb34	1/d	10d a period, 2peirods	Sham acupuncture	Deep insertion at nonacupoints	1/d	10d a period, 2peirods
					Flunarizine Hydrochloride	5mg	qn	4w
Zhang,2016a	acupuncture	GB20, Sj5, SP6	1/d	10times a period,	Ibuprofen;Flunarizine Hydrochloride	0.3g; 10mg	2/d for acute periods; qn	10times a period,

Study	Intervention	Acupoints	Frequency	Duration of treatment	Comparison	Single dose / Acupoints	Frequency	Duration of treatment
				2periods				2periods
Zhang,2016b	acupuncture	GB20, ST8, EX-HN5, GB34, LR3, KI3, GB8, SJ5, GB5, GB41	1/d	1m	Betahistine mesilate	6mg	3/d	1m
Zhao,2011	acupuncture	SJ23, SJ5, GB20, GB40/PC3, PC6, LR3, LR5/LI4, LI6, ST8, ST36	4/w	4w	Sham acupuncture	Deep insertion at nonacupoints	4/w	4w
Zhao,2014	acupuncture	GB8, GB5, GB7, Piantouxue	3/w	1w	Flunarizine Hydrochloride	10mg	qn	1w
Zheng,2013	acupuncture	GB20, GB34, SJ5, SJ17	1/d	5/w, 4w	Flunarizine Hydrochloride	10mg	2/d	4w
Zheng,2016	acupuncture	EX-HN5, GB7, ST8, GB8, SJ23, GB5, GB15, GB20, GB34, GB41	1/d	7d a period, 2periods	Flunarizine Hydrochloride	5mg	qn	7d a period, 2periods
Zhou,2017	acupuncture	GV20, GB20, ST8, SP6, LR3, KI3, SJ5	1/d	2w	Flunarizine Hydrochloride	10mg	qn	2w

Note: Abbreviations: T, intervention group; C, comparison group.

Supplementary Tables 2. Acupuncture vs. no treatment.

Outcome or Subgroup	Studies	Participants	Statistical Method	Effect Estimate
1.1 Days of attack	1		Mean Difference (IV, Fixed, 95% CI)	Subtotals only
1.1.1 Immediate effects	0	0	Mean Difference (IV, Fixed, 95% CI)	Not estimable
1.1.2 Up to 1 month after treatment	1	221	Mean Difference (IV, Fixed, 95% CI)	-1.40[-2.38, -0.42]
1.1.3 1 to 3 months after treatment	0	0	Mean Difference (IV, Fixed, 95% CI)	Not estimable
1.1.4 3 to 6 months after treatment	0	0	Mean Difference (IV, Fixed, 95% CI)	Not estimable
1.1.5 > 6 months after treatment	0	0	Mean Difference (IV, Fixed, 95% CI)	Not estimable
1.2 Attack frequency	1	221	Mean Difference (IV, Fixed, 95% CI)	-0.80 [-1.12, -0.48]
1.2.1 Immediate effects	0	0	Mean Difference (IV, Fixed, 95% CI)	Not estimable
1.2.2 Up to 1 month after treatment	1	221	Mean Difference (IV, Fixed, 95% CI)	-0.80 [-1.12, -0.48]
1.2.3 1 to 3 months after treatment	0	0	Mean Difference (IV, Fixed, 95% CI)	Not estimable
1.2.4 3 to 6 months after treatment	0	0	Mean Difference (IV, Fixed, 95% CI)	Not estimable
1.2.5 > 6 months after treatment	0	0	Mean Difference (IV, Fixed, 95% CI)	Not estimable

1.2 Attack frequency	1	221	Mean Difference (IV, Fixed, 95% CI)	-0.80 [-1.12, -0.48]

Supplementary Tables 3. Subgroup analysis-Acupuncture vs. Medication.

Outcome Title	Number of studies	Number of patients	Statistical Method	Effect Estimate	Test for overall effect P-value	Heterogeneity;I²
Effective rates: up to 1 month after treatment						
Sample size						
< median sample	12	702	Risk Ratio (M-H, Random, 95% CI)	1.22 [1.11, 1.35]	P<0.0001	46%
≥ median sample	9	896	Risk Ratio (M-H, Random, 95% CI)	1.18 [1.09, 1.28]	P<0.0001	50%
Treatment time						
< 1 month	7	483	Risk Ratio (M-H, Random, 95% CI)	1.30 [1.08, 1.58]	0.006	75%

≥ 1 month	14	1115	Risk Ratio (M-H, Random, 95% CI)	1.17 [1.12, 1.23]	P<0.0001	0%
Adequacy of concealment						
None or unclear adequacy	18	1402	Risk Ratio (M-H, Random, 95% CI)	1.21 [1.13, 1.29]	P<0.0001	46%
Unambigously adequate concealment	3	196	Risk Ratio (M-H, Random, 95% CI)	1.12 [0.99, 1.27]	0.07	16%
Type of medication						
Aspirin	2	120	Risk Ratio (M-H, Random, 95% CI)	1.08 [0.93, 1.27]	0.32	0%
Combined medication	6	509	Risk Ratio (M-H, Random, 95% CI)	1.18 [1.02, 1.37]	0.03	61%
Flunarizine Hydrochloride	10	722	Risk Ratio (M-H, Random, 95% CI)	1.22 [1.13, 1.33]	P<0.0001	35%
Honglongzhentong Tablet	1	120	Risk Ratio (M-H, Random, 95% CI)	1.37 [1.17, 1.61]	0.0001	Not applicable
Indometacin	1	63	Risk Ratio (M-H, Random, 95% CI)	1.28 [0.99, 1.64]	0.06	Not applicable
Naproxen	1	64	Risk Ratio (M-H, Random, 95% CI)	1.11 [0.96, 1.28]	0.17	Not applicable

Effective rates: 3 to 6 month after treatment						
Sample size						
< median sample	2	120	Risk Ratio (M-H, Random, 95% CI)	1.23 [1.06, 1.43]	0.007	0%
≥ median sample	4	345	Risk Ratio (M-H, Random, 95% CI)	1.17 [0.96, 1.42]	0.12	67%
Treatment time						
< 1 month	1	125	Risk Ratio (M-H, Random, 95% CI)	1.00 [0.83, 1.21]	0.96	Not applicable
≥ 1 month	5	340	Risk Ratio (M-H, Random, 95% CI)	1.23 [1.07, 1.42]	0.005	49%
Type of medication						
Flunarizine Hydrochloride	5	385	Risk Ratio (M-H, Random, 95% CI)	1.22 [1.05, 1.42]	0.008	53%
Gabapentin	1	80	Risk Ratio (M-H, Random, 95% CI)	1.03 [0.85, 1.25]	0.76	Not applicable
VAS scores: up to 1 month after treatment						
Sample size						

< median sample	4	242	Mean Difference (IV, Random, 95% CI)	-1.12[-1.81, -0.43]	0.002	66%
≥ median sample	8	705	Mean Difference (IV, Random, 95% CI)	-1.26[-1.67, -0.84]	P<0.0001	88%
Treatment time						
< 1 month	6	511	Mean Difference (IV, Random, 95% CI)	-1.18[-1.84, -0.53]	0.0004	91%
≥ 1 month	6	436	Mean Difference (IV, Random, 95% CI)	-1.27[-1.56, -0.99]	P<0.0001	46%
Adequacy of concealment						
None or unclear adequacy	10	813	Mean Difference (IV, Random, 95% CI)	-1.29[-1.69, -0.90]	P<0.0001	85%
Unambigously adequate concealment	2	134	Mean Difference (IV, Random, 95% CI)	-0.80[-1.95, 0.34]	0.17	86%
Type of medication						
Aspirin	1	60	Mean Difference (IV, Random, 95% CI)	-1.40[-2.39, -0.41]	0.005	Not applicable
Carbamazepine	1	128	Mean Difference (IV, Random, 95% CI)	-1.79[-2.01, -1.57]	P<0.0001	Not applicable

Flunarizine Hydrochloride	8	605	Mean Difference (IV, Random, 95% CI)	-1.29[-1.61, -0.97]	P<0.0001	70%
Combined medication	2	154	Mean Difference (IV, Random, 95% CI)	-0.49[-2.71, 1.72]	0.66	96%
Adverse events						
Sample size						
< median sample	5	275	Risk Difference (M-H, Random, 95% CI)	-0.12[-0.29, 0.06]	0.2	90%
≥ median sample	9	970	Risk Difference (M-H, Random, 95% CI)	-0.18[-0.30, -0.06]	0.004	95%
Treatment time						
< 1 month	8	711	Risk Difference (M-H, Random, 95% CI)	-0.15[-0.27, -0.03]	0.02	91%
≥ 1 month	6	534	Risk Difference (M-H, Random, 95% CI)	-0.17[-0.34, 0.01]	0.06	96%
Adequacy of concealment						
None or unclear adequacy	9	806	Risk Difference (M-H, Random, 95% CI)	-0.16[-0.27, -0.05]	0.004	92%

Unambigously adequate concealment	5	439	Risk Difference (M-H, Random, 95% CI)	-0.14[-0.36, 0.07]	0.19	95%
Type of medication						
Valproic acid	1	93	Risk Difference (M-H, Random, 95% CI)	-0.43[-0.58, -0.29]	P<0.0001	Not applicable
Combined medication	2	154	Risk Difference (M-H, Random, 95% CI)	-0.07[-0.29, 0.15]	0.54	88%
Flunarizine Hydrochloride	7	768	Risk Difference (M-H, Random, 95% CI)	-0.13[-0.23, -0.03]	0.01	88%
Topiramate	1	66	Risk Difference (M-H, Random, 95% CI)	-0.61[-0.79, -0.43]	P<0.0001	Not applicable
Sumatriptan	1	31	Risk Difference (M-H, Random, 95% CI)	-0.29[-0.63, 0.05]	0.09	Not applicable
Naproxen	1	64	Risk Difference (M-H, Random, 95% CI)	0.06[-0.04, 0.16]	0.22	Not applicable
Betahistine mesilate	1	69	Risk Difference (M-H, Random, 95% CI)	0.00[-0.05, 0.05]	1.00	Not applicable
Days of attack: up to 1 month after treatment						
Treatment time						

< 1 month	1	64	Mean Difference (IV, Random, 95% CI)	-0.59[-1.04, -0.14]	0.01	Not applicable
≥ 1 month	2	120	Mean Difference (IV, Random, 95% CI)	-0.98[-4.13, 2.16]	0.54	97%
Adequacy of concealment						
None or unclear adequacy	2	122	Mean Difference (IV, Random, 95% CI)	-1.58[-3.50, 0.35]	0.11	97%
Unambigously adequate concealment	1	62	Mean Difference (IV, Random, 95% CI)	0.65[-0.31, 1.61]	0.18	Not applicable

Supplementary Tables 3. Subgroup analysis-Acupuncture vs. Medication.

Outcome Title	Number of studies	Number of patients	Statistical Method	Effect Estimate	Test for overall effect P-value	Heterogeneity; I^2
Effective rates: up to 1 month after treatment						
Sample size						
< median sample	12	702	Risk Ratio (M-H, Random, 95% CI)	1.22 [1.11, 1.35]	P<0.0001	46%
≥ median sample	9	896	Risk Ratio (M-H, Random, 95% CI)	1.18 [1.09, 1.28]	P<0.0001	50%

Treatment time						
< 1 month	7	483	Risk Ratio (M-H, Random, 95% CI)	1.30 [1.08, 1.58]	0.006	75%
≥ 1 month	14	1115	Risk Ratio (M-H, Random, 95% CI)	1.17 [1.12, 1.23]	P<0.0001	0%
Adequacy of concealment						
None or unclear adequacy	18	1402	Risk Ratio (M-H, Random, 95% CI)	1.21 [1.13, 1.29]	P<0.0001	46%
Unambigously adequate concealment	3	196	Risk Ratio (M-H, Random, 95% CI)	1.12 [0.99, 1.27]	0.07	16%
Type of medication						
Aspirin	2	120	Risk Ratio (M-H, Random, 95% CI)	1.08 [0.93, 1.27]	0.32	0%
Combined medication	6	509	Risk Ratio (M-H, Random, 95% CI)	1.18 [1.02, 1.37]	0.03	61%
Flunarizine Hydrochloride	10	722	Risk Ratio (M-H, Random, 95% CI)	1.22 [1.13, 1.33]	P<0.0001	35%
Honglongzhentong Tablet	1	120	Risk Ratio (M-H, Random, 95% CI)	1.37 [1.17, 1.61]	0.0001	Not applicable
Indometacin	1	63	Risk Ratio (M-H, Random, 95% CI)	1.28 [0.99, 1.64]	0.06	Not applicable
Naproxen	1	64	Risk Ratio (M-H, Random, 95% CI)	1.11 [0.96, 1.28]	0.17	Not applicable
			Effective rates: 3 to 6 month after treatment			
Sample size						
< median sample	2	120	Risk Ratio (M-H, Random, 95% CI)	1.23 [1.06, 1.43]	0.007	0%

≥ median sample	4	345	Risk Ratio (M-H, Random, 95% CI)	1.17 [0.96, 1.42]	0.12	67%
Treatment time						
< 1 month	1	125	Risk Ratio (M-H, Random, 95% CI)	1.00 [0.83, 1.21]	0.96	Not applicable
≥ 1 month	5	340	Risk Ratio (M-H, Random, 95% CI)	1.23 [1.07, 1.42]	0.005	49%
Type of medication						
Flunarizine Hydrochloride	5	385	Risk Ratio (M-H, Random, 95% CI)	1.22 [1.05, 1.42]	0.008	53%
Gabapentin	1	80	Risk Ratio (M-H, Random, 95% CI)	1.03 [0.85, 1.25]	0.76	Not applicable
VAS scores: up to 1 month after treatment						
Sample size						
< median sample	4	242	Mean Difference (IV, Random, 95% CI)	-1.12[-1.81, -0.43]	0.002	66%
≥ median sample	8	705	Mean Difference (IV, Random, 95% CI)	-1.26[-1.67, -0.84]	P<0.0001	88%
Treatment time						
< 1 month	6	511	Mean Difference (IV, Random, 95% CI)	-1.18[-1.84, -0.53]	0.0004	91%
≥ 1 month	6	436	Mean Difference (IV, Random, 95% CI)	-1.27[-1.56, -0.99]	P<0.0001	46%
Adequacy of concealment						

None or unclear adequacy	10	813	Mean Difference (IV, Random, 95% CI)	-1.29[-1.69, -0.90]	P<0.0001	85%
Unambigously adequate concealment	2	134	Mean Difference (IV, Random, 95% CI)	-0.80[-1.95, 0.34]	0.17	86%
Type of medication						
Aspirin	1	60	Mean Difference (IV, Random, 95% CI)	-1.40[-2.39, -0.41]	0.005	Not applicable
Carbamazepine	1	128	Mean Difference (IV, Random, 95% CI)	-1.79[-2.01, -1.57]	P<0.0001	Not applicable
Flunarizine Hydrochloride	8	605	Mean Difference (IV, Random, 95% CI)	-1.29[-1.61, -0.97]	P<0.0001	70%
Combined medication	2	154	Mean Difference (IV, Random, 95% CI)	-0.49[-2.71, 1.72]	0.66	96%
Adverse events						
Sample size						
< median sample	5	275	Risk Difference (M-H, Random, 95% CI)	-0.12[-0.29, 0.06]	0.2	90%
≥ median sample	9	970	Risk Difference (M-H, Random, 95% CI)	-0.18[-0.30, -0.06]	0.004	95%
Treatment time						
< 1 month	8	711	Risk Difference (M-H, Random, 95% CI)	-0.15[-0.27, -0.03]	0.02	91%
≥ 1 month	6	534	Risk Difference (M-H, Random, 95% CI)	-0.17[-0.34, 0.01]	0.06	96%

Adequacy of concealment						
None or unclear adequacy	9	806	Risk Difference (M-H, Random, 95% CI)	-0.16[-0.27, -0.05]	0.004	92%
Unambigously adequate concealment	5	439	Risk Difference (M-H, Random, 95% CI)	-0.14[-0.36, 0.07]	0.19	95%
Type of medication						
Valproic acid	1	93	Risk Difference (M-H, Random, 95% CI)	-0.43[-0.58, -0.29]	P<0.0001	Not applicable
Combined medication	2	154	Risk Difference (M-H, Random, 95% CI)	-0.07[-0.29, 0.15]	0.54	88%
Flunarizine Hydrochloride	7	768	Risk Difference (M-H, Random, 95% CI)	-0.13[-0.23, -0.03]	0.01	88%
Topiramate	1	66	Risk Difference (M-H, Random, 95% CI)	-0.61[-0.79, -0.43]	P<0.0001	Not applicable
Sumatriptan	1	31	Risk Difference (M-H, Random, 95% CI)	-0.29[-0.63, 0.05]	0.09	Not applicable
Naproxen	1	64	Risk Difference (M-H, Random, 95% CI)	0.06[-0.04, 0.16]	0.22	Not applicable
Betahistine mesilate	1	69	Risk Difference (M-H, Random, 95% CI)	0.00[-0.05, 0.05]	1.00	Not applicable
Days of attack: up to 1 month after treatment						

Treatment time						
< 1 month	1	64	Mean Difference (IV, Random, 95% CI)	-0.59[-1.04, -0.14]	0.01	Not applicable
≥ 1 month	2	120	Mean Difference (IV, Random, 95% CI)	-0.98[-4.13, 2.16]	0.54	97%
Adequacy of concealment						
None or unclear adequacy	2	122	Mean Difference (IV, Random, 95% CI)	-1.58[-3.50, 0.35]	0.11	97%
Unambigously adequate concealment	1	62	Mean Difference (IV, Random, 95% CI)	0.65[-0.31, 1.61]	0.18	Not applicable

Supplementary Tables 4. Subgroup analysis-Acupuncture vs. sham acupuncture.

Outcome Title	Number of studies	Number of patients	Statistical Method	Effect Estimate	Test for overall effect P-value	Heterogeneity;I²
VAS scores: up to 1 month after treatment						
Sample size						
< median sample	8	336	Mean Difference (IV, Random, 95% CI)	-1.46 [-2.31, -0.62]	0.0007	82%
≥ median sample	1	73	Mean Difference (IV,	-2.06 [-2.32, -	P<0.0001	Not applicabl

			Random, 95% CI)	1.80]		e
Treatment time						
< 1 month	1	28	Mean Difference (IV, Random, 95% CI)	-1.54 [-3.76, 0.68]	0.17	Not applicabl e
≥ 1 month	8	381	Mean Difference (IV, Random, 95% CI)	-1.56 [-2.24, -0.89]	P<0.0001	84%
Adequacy of concealment						
None or unclear adequacy	5	242	Mean Difference (IV, Random, 95% CI)	-1.39 [-2.63, -0.15]	0.03	90%
Unambigously adequate concealment	4	167	Mean Difference (IV, Random, 95% CI)	-1.94 [-2.24, -1.63]	P<0.0001	7%
Acupuncture method						
Deep insertion at nonacupoints	8	359	Mean Difference (IV, Random, 95% CI)	-1.75 [-2.38, -1.11]	P<0.0001	79%
Superficial insertion at nonacupoints	1	50	Mean Difference (IV, Random, 95% CI)	-0.20 [-1.20, 0.80]	0.69	Not applicabl e
Days of attack: up to 1 month after treatment						

Sample size						
< median sample	6	262	Mean Difference (IV, Fixed, 95% CI)	-1.57 [-2.43, -0.70]	0.0004	38%
≥ median sample	1	226	Mean Difference (IV, Fixed, 95% CI)	0.20 [-0.72, 1.12]	0.02	61%
Treatment time						
< 1 month	6	445	Mean Difference (IV, Random, 95% CI)	-1.36 [-2.73, 0.01]	0.05	67%
≥ 1 month	1	43	Mean Difference (IV, Random, 95% CI)	-1.29 [-3.04, 0.46]	0.03	61%
Adequacy of concealment						
None or unclear adequacy	4	184	Mean Difference (IV, Random, 95% CI)	-1.88 [-3.31, -0.46]	0.009	44%
Unambigously adequate concealment	3	304	Mean Difference (IV, Random, 95% CI)	-0.56 [-2.18, 1.06]	0.5	59%
Acupuncture method						
Deep insertion at nonacupoints	2	70	Mean Difference (IV, Random, 95% CI)	-0.99 [-2.22, 0.24]	0.12	0%

Superficial insertion at acupoints	3	142	Mean Difference (IV, Random, 95% CI)	-1.69 [-3.34, -0.05]	0.04	38%
Superficial insertion at nonacupoints	3	142	Mean Difference (IV, Random, 95% CI)	-1.69 [-3.34, -0.05]	0.41	87%
Days of attack: 1 to 3 month after treatment						
Adequacy of concealment						
None or unclear adequacy	2	114	Mean Difference (IV, Random, 95% CI)	-2.69 [-5.71, 0.33]	0.08	66%
Unambigously adequate concealment	3	81	Mean Difference (IV, Random, 95% CI)	0.98 [-0.78, 2.74]	0.27	38%
Acupuncture method						
Deep insertion at nonacupoints	1	27	Mean Difference (IV, Random, 95% CI)	-0.58 [-2.73, 1.57]	0.6	Not applicable
Superficial insertion at acupoints	2	90	Mean Difference (IV, Random, 95% CI)	0.04 [-3.14, 3.21]	0.98	60%
Superficial insertion at nonacupoints	2	78	Mean Difference (IV, Random, 95% CI)	-1.16 [-7.33, 5.01]	0.71	92%
Attack frequency: up to 1 month after treatment						

Sample size						
< median sample	7	237	Mean Difference (IV, Random, 95% CI)	-0.38 [-0.90, 0.14]	0.15	43%
≥ median sample	2	326	Mean Difference (IV, Random, 95% CI)	-0.65 [-1.72, 0.43]	0.24	94%
Treatment time						
< 1 month	3	164	Mean Difference (IV, Fixed, 95% CI)	-1.19 [-1.56, -0.82]	P<0.0001	0%
≥ 1 month	6	399	Mean Difference (IV, Fixed, 95% CI)	-0.13 [-0.39, 0.13]	0.32	35%
Adequacy of concealment						
None or unclear adequacy	2	107	Mean Difference (IV, Random, 95% CI)	-0.81 [-1.41, -0.21]	0.009	0%
Unambigously adequate concealment	7	456	Mean Difference (IV, Random, 95% CI)	-0.37 [-0.97, 0.22]	0.21	78%
Acupuncture method						
Deep insertion at nonacupoints	2	64	Mean Difference (IV, Random, 95% CI)	-1.13 [-2.14, -0.12]	0.03	0%

Superficial insertion at acupoints	3	126	Mean Difference (IV, Random, 95% CI)	-0.47 [-0.98, 0.04]	0.07	0%
Superficial insertion at nonacupoints	4	373	Mean Difference (IV, Random, 95% CI)	-0.36 [-1.18, 0.46]	0.39	89%
Attack frequency: 1 to 3 month after treatment						
Sample size						
< median sample	4	140	Mean Difference (IV, Random, 95% CI)	-1.80 [-5.64, 2.03]	0.36	99%
≥ median sample	1	100	Mean Difference (IV, Random, 95% CI)	-0.60 [-0.99, -0.21]	0.03	Not applicable
Treatment time						
< 1 month	1	100	Mean Difference (IV, Random, 95% CI)	-0.60 [-0.99, -0.21]	0.03	Not applicable
≥ 1 month	4	140	Mean Difference (IV, Random, 95% CI)	-1.80 [-5.64, 2.03]	0.36	99%
Adequacy of concealment						
None or unclear adequacy	2	86	Mean Difference (IV,	-4.19 [-11.44,	0.26	99%

			Random, 95% CI)	3.06]		
Unambigously adequate concealment	3	154	Mean Difference (IV, Random, 95% CI)	0.11 [-0.87, 1.09]	0.83	78%
Acupuncture method						
Superficial insertion at acupoints	3	112	Mean Difference (IV, Random, 95% CI)	-2.47 [-7.57, 2.64]	0.34	99%
Superficial insertion at nonacupoints	2	128	Mean Difference (IV, Random, 95% CI)	-0.33 [-1.07, 0.42]	0.39	56%
MSQ - role function-restrictive: up to 1 month after treatment						
Treatment time						
< 1 month	6	183	Mean Difference (IV, Random, 95% CI)	11.31 [7.02, 15.60]	P<0.0001	95%
≥ 1 month	1	50	Mean Difference (IV, Random, 95% CI)	14.20 [3.70, 24.70]	0.008	Not applicable
Adequacy of concealment						
None or unclear adequacy	4	182	Mean Difference (IV, Random, 95% CI)	14.92 [6.71, 23.12]	0.0004	88%
Unambigously adequate	3	94	Mean Difference (IV,	6.87 [1.09, 12.64]	0.02	94%

concealment			Random, 95% CI)			
Acupuncture method						
Deep insertion at nonacupoints	6	226	Mean Difference (IV, Random, 95% CI)	11.31 [7.02, 15.60]	P<0.0001	95%
Superficial insertion at nonacupoints	1	50	Mean Difference (IV, Random, 95% CI)	14.20 [3.70, 24.70]	0.008	Not applicabl e
MSQ - role function-preventive: up to 1 month after treatment						
Treatment time						
< 1 month	6	226	Mean Difference (IV, Random, 95% CI)	10.10 [0.82, 19.38]	0.03	85%
≥ 1 month	1	50	Mean Difference (IV, Random, 95% CI)	8.80 [-2.34, 19.94]	0.12	Not applicabl e
Adequacy of concealment						
None or unclear adequacy	4	182	Mean Difference (IV, Random, 95% CI)	11.41 [-1.12, 23.94]	0.07	90%
Unambigously adequate concealment	3	94	Mean Difference (IV, Random, 95% CI)	6.54 [0.93, 12.14]	0.02	0%

Acupuncture method						
Deep insertion at nonacupoints	6	226	Mean Difference (IV, Random, 95% CI)	11.31 [7.02, 15.60]	0.03	85%
Superficial insertion at nonacupoints	1	50	Mean Difference (IV, Random, 95% CI)	14.20 [3.70, 24.70]	0.12	Not applicable
MSQ - emotional function: up to 1 month after treatment						
Treatment time						
< 1 month	6	226	Mean Difference (IV, Random, 95% CI)	9.12 [-0.25, 18.49]	0.06	82%
≥ 1 month	1	50	Mean Difference (IV, Random, 95% CI)	17.80 [5.15, 30.45]	0.006	Not applicable
Adequacy of concealment						
None or unclear adequacy	4	182	Mean Difference (IV, Random, 95% CI)	13.93 [3.39, 24.47]	0.01	81%
Unambigously adequate concealment	3	94	Mean Difference (IV, Random, 95% CI)	3.20 [-2.77, 9.17]	0.29	0%
Acupuncture method						

Deep insertion at nonacupoints	6	226	Mean Difference (IV, Random, 95% CI)	9.12 [-0.25, 18.49]	0.06	82%
Superficial insertion at nonacupoints	1	50	Mean Difference (IV, Random, 95% CI)	17.80 [5.15, 30.45]	0.006	Not applicabl e
